# Sustainable chitosan and medicinal plant oils as natural edible coatings for postharvest quality preservation of guava fruits (*Psidium guajava* L.)

**DOI:** 10.1371/journal.pone.0342650

**Published:** 2026-03-18

**Authors:** Assiya Ansabayeva, Nazmy A. Abdel Ghany, Shams A. Hussein, Karim M. Hassan, Ahmed N. Abdelhamid, Mohamed K. Abou El-Nasr, Ahmed Bondok, Nazih Y. Rebouh, Mostafa Abdelkader, Mohamed A. Nasser

**Affiliations:** 1 Department of Agronomy, Faculty of Agriculture, Akhmet Baitursynuly Kostanay Regional University, Kazakhstan; 2 Department of Horticulture, Faculty of Agriculture, Ain Shams University, Cairo, Egypt; 3 Department of Plant Pathology, Faculty of Agriculture, Ain Shams University, Cairo, Egypt; 4 Department of Environmental Management, Institute of Environmental Engineering, RUDN University, Moscow, Russia; 5 Horticulture Department, Faculty of Agriculture, Sohag University, Sohag, Egypt; Amity University Noida, INDIA

## Abstract

Postharvest loss of tropical fruits remains a significant challenge for food security and sustainability, as their delicate texture and high metabolic activity make them highly susceptible to rapid deterioration through handling and distribution. This study investigates the application of an edible coating technique to reduce loss, deterioration, and spoilage, and to increase the shelf-life of guava fruit. The fruits were immersed in chitosan and edible oils (moringa, lemongrass, marjoram, and rosemary). Chitosan (2%) and 1–2% moringa oil extended the shelf life of fruits by up to 24 days. Moringa oil at 1–2% mitigated the loss in fruit weight compared to 1% chitosan after the 24th day of storage, and the fruits had superior quality attributes (TSS, vitamin C, sugar content). The findings show that 2% chitosan and 1–2% moringa oil were the most effective treatments, reducing weight loss to about 10–25% compared to nearly 50% in untreated fruits and maintaining overall fruit quality. These treatments boosted peroxidase (POD) activity, reaching a peak of 76.47 U/g and keeping hydrogen peroxide (H_2_O_2_) levels low at 24.25 µmol g^-^¹ FW, signalling strong protection against oxidative stress. On the chemical side, they maintained higher total soluble solids (13.17 °Brix), total sugars (11.36%), and vitamin C (32.49 mg/100 mL), while keeping acidity lower (0.82%). By comparison, lemongrass and marjoram oil treatments were far less effective, showing faster weight loss and oxidative damage levels similar to those of the control group. Chitosan and oil coatings substantially reduce bacteria and yeasts/moulds on guava fruits, and moringa oil treatment improved physio-biochemical characteristics and reduced postharvest disease spoilage. The results highlight that natural coatings, particularly chitosan and moringa oil, not only preserved the physio-biochemical quality of guava but also offered a sustainable, biodegradable solution that can help reduce food loss, limit reliance on synthetic chemicals, and support environmentally responsible postharvest management practices.

## 1. Introduction

Reducing agricultural production losses is critical challenge to meet the rising food security of a global population nearing eight billion [1,2]. Guava is an economically important crop in tropical and subtropical regions [3] and is valued for its high nutritional and medicinal properties, which are used to treat several diseases [4]. Guava has outstanding qualities, although postharvest storage is difficult [5–8]. As a climacteric fruit, it has a very short shelf life of three to four days at room temperature due to rapid respiration and ethylene production, leading to higher postharvest than preharvest losses [9,10]. Fruit senescence involves biochemical and physiological changes that reduce quality and nutritional value, making the control of deterioration essential for maintaining antioxidant activity and improving the nutritional quality of fruits and vegetables [2,11,12].

As guava ages, it is prone to weight loss and microbial infections, which accelerate decay and reduce marketability and shelf life [13,14]. Applying essential oils and chitosan can control postharvest losses and maintain fruit quality [15]. Essential oils, natural antioxidants with antimicrobial and biodegradable properties [16,17], leave no residue on fruits. Their antibacterial activity is linked to plant-derived secondary metabolites {Citation} whose hydrophobic components disrupt microbial cell membranes, causing metabolic damage and cell death [18,19].

The edible coating technique refers to a thin layer made from safe, edible substances that is applied to fruits and vegetables to help prolong their shelf life [20]. They are biodegradable, safe to consume, and often made from polysaccharides, proteins, or lipids, offering an eco-friendly alternative to synthetic materials [21,22]. These coatings help preserve food quality, ensure safety, and reduce waste [23], while meeting standards such as appealing sensory properties, effective barriers to moisture and gases, mechanical strength, biochemical and microbiological stability, safety, and cost-effectiveness [23,24]. They can also be enhanced with antioxidants, flavorings, or sweeteners to create active packaging that improves nutritional value and sensory appeal [25,26].

Recent advances in edible coating technology emphasize the use of biopolymer-based composite systems, particularly polysaccharide-based materials such as chitosan, alginate, pullulan, xanthan gum, starch, and natural gums, owing to their excellent film-forming ability, biodegradability, biocompatibility, and capacity to act as carriers for bioactive compounds [27–32]. Polysaccharides play a crucial role in edible coatings by forming cohesive matrices that regulate gas exchange and moisture migration, thereby delaying senescence and quality degradation in fresh produce [29].

The mechanism of action of edible coatings primarily involves the formation of a semipermeable barrier on the fruit surface, which reduces transpiration, slows oxygen diffusion, lowers the respiration rate, suppresses ethylene biosynthesis, and delays enzymatic reactions related to softening, browning, and oxidative stress [33,34]. Additionally, the incorporation of functional bioactive agents such as essential oils, plant extracts, and nano-emulsions transforms conventional edible coatings into active packaging systems capable of providing antioxidant and antimicrobial protection [34,35].

Edible coatings extend fruit shelf life by preserving appearance, firmness, and moisture. They act as barriers to moisture and gases, slow lipid oxidation, regulate enzymes, and reduce microbial spoilage [36]. Often enriched with antimicrobial agents like organic acids, essential oils, or polypeptides, they inhibit microbial growth, slow browning, prevent softening, and retain natural aroma, flavor, and color [23,37–40]. Overall, edible coatings protect against contamination, limit deterioration, reduce moisture loss and oxidation, and extend storage life [41–43].

Composite edible coatings have shown superior performance compared to single-component systems due to synergistic interactions between polymers and functional additives, resulting in improved mechanical strength, barrier efficiency, and controlled release of antimicrobial compounds [44,45]. In particular, chitosan–sodium alginate-based antimicrobial composite coatings functionalized with nanoemulsions or plant-derived extracts have been reported to significantly extend the shelf life of guava and other climacteric fruits by reducing weight loss, microbial load, enzymatic activity, and oxidative deterioration during storage [20,46].

Several studies demonstrate that pre-harvest application of essential oils helps preserve guava quality and extend shelf life by reducing weight loss, peroxidase activity, mould growth, and color changes. Notable essential oils used as edible coatings include mustard, olive, coconut, almond, grape seed [47]; jojoba oil [48]; essential oils of eucalyptus, tulsi, and neem [49]; cinnamon essential oil, coconut oil, rosemary oil, and moringa [50,51]; and the essential oil of peppermint [52] as edible coating materials. Additionally, chitosan coatings prevent moisture loss, gas exchange, and microbial invasion, maintaining guava firmness, acidity, and nutritional value [53,54]. These combined procedures provide a comprehensive solution for postharvest preservation, ensuring the long-term storage of guava fruits, particularly under cold temperatures. These findings align with recent trends in sustainable edible packaging, which highlight the integration of biobased polymers, natural extracts, and nano-delivery systems as an effective strategy to enhance postharvest quality, extend shelf life, and support bioeconomy approaches in fruit preservation [55,56].

This study aims to evaluate how chitosan and essential oils as edible coatings can improve postharvest quality, prevent decay, and extend guava shelf life.

## 2. Materials and methods

### 2.1. Plant material

The winter crop of ‘Etmani’ guava trees was harvested from 14-year-old trees grown in clayey loam soil on a private farm in Qalyubia Governorate, Egypt. The fruits were harvested at the ripening stage (yellowish green) in the second week of February, according to Mercado Silva et al. [57]. The fruits were divided into groups of similar sizes, with no apparent signs of infection or damage. The fruits were soaked in 1% sodium hypochlorite solution for 3 minutes to sterilise them, then washed with distilled water and dried at room temperature.

### 2.2. Experimental design

**Fig 1:**
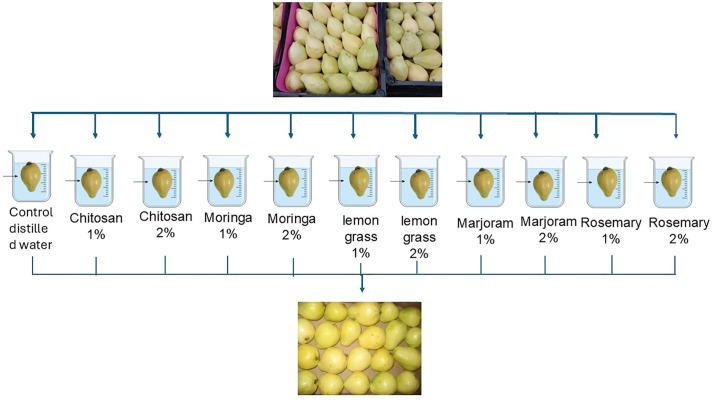
Schematic representation of the edible coating treatments applied to guava fruits, including chitosan (1% and 2%), moringa extract (1% and 2%), lemongrass extract (1% and 2%), marjoram extract (1% and 2%), and rosemary extract (1% and 2%), with distilled water (control) for the evaluation of postharvest quality attributes.

The experiment was performed in a completely randomized design (CRD). The fruits were divided among eleven treatments: Distilled water (control), chitosan 1–2%, Moringa oil 1–2%, lemongrass oil 1–2%, Marjoram 1–2%, Rosemary 1–2% (Fig 1). Chitosan (Sigma-Aldrich, St. Louis, MO, USA), medicinal plant Oils (from the National Research Center. Giza, Egypt) Each treatment was replicated five times with 20 fruits per replicate.

For 5 minutes, the guava fruits were submerged in a treatment solution containing 0.05% (v/v) Tween-80 to improve their wettability and surface adhesion. The fruits were dried at room temperature until their surfaces were completely free of any remaining moisture. Every replicate was enclosed in a cardboard box. After that, the boxes were kept at 90% relative humidity and 8 ± 1°C. Until half of the fruit is ruined, the treatment is kept in storage. To assess the effects of edible coating treatments on the physical and chemical characteristics and on diseases that develop, identify the type of disease, and gauge the effectiveness of the treatment applied to guava fruits, each treatment was assessed at harvest and then every 4 days thereafter.

### 2.3. Physical measurements

Fruit weight loss (%) was measured by calculating the reduction percentage in mass from the initial to the final day of storage [58]. Firmness (N/cm²) was determined using a fruit texture analyser (model GS- serial number FTA2) [9]. Panellists assessed the organoleptic rating attribute with a score of 6.00 (good), 7.00 (very good), 8.00 (excellent), and 9.00 (outstanding) according to Pereira L. et al. [59].

### 2.4. Chemical measurements

Total soluble solids (TSS) were determined in fruit juice using a hand refractometer v. HR110 [60]. Titratable acidity (TA%) was measured in 10 ml of fruit juice as a percentage of citric acid, determined by titration with 0.1 N sodium hydroxide solution, using 1% phenolphthalein as an indicator [11,61]. The TSS/TA ratio was calculated by dividing the acidity by the TSS values [60]. Vitamin C (mg/100 ml of the juice) was determined using 2,6-dichlorophenol indophenol in ten pulp mixed with 3% oxalic acid [62]. Total sugars (%), reduced sugars (%), and non-reduced sugars (%) were determined by the iodometric titration method [63].

### 2.5. Oxidative stress markers

The peroxidase activity (POD) assay was performed to determine its ability to convert guaiacol into tetra guaiacol. In brief, 1 gram of fruit was crushed in 4 millilitres of phosphate extraction buffer. The reaction mixture contained 2.9 mL of 100 mM phosphate buffer (pH 7.0), 20.1 mM guaiacol, 10 mM H_2_O_2_, and 0.1 mL of enzyme extract. The absorbance rise was measured by injecting H_2_O_2_ at 470 nm for 3 minutes [64,65]. The content of hydrogen peroxide (H_2_O_2_) was tested with 500 mg of fruit samples and 3 mL of 1% (w/v) tri-chloroacetic acid (TCA). The homogenate was centrifuged at 10,000 rpm at 4°C for 10 minutes. Following that, 0.75 mL of supernatant was combined with 0.75 mL of 10 mm K-phosphate buffer (pH 7.0), 1.5 mL of 1M KI, and 0.75 mL of the supernatant. A standard calibration curve was compared to the observed absorbance of H_2_O_2_ at 390 nm [66]. The H_2_O_2_ concentration was estimated using a standard curve ranging from 0 to 15 nmol mL^-1^.

### 2.6. Fruit postharvest disease percentage (F.P.D. %)

The fraction of disordered fruits, which includes all spoiled fruits caused by rots and fungal infections, was assessed. Each treatment in the experiment was repeated three times with 20 fruits, and the findings were expressed as a percentage of the beginning fruit number. Faults were identified by calculating the number of decayed fruits at the harvesting date expressed as a percentage of the initial number of fruits, based on the formula: F.P.D.% = ((A/ B) × 100), where (A) is the number of decayed fruits at the time of sampling and (B) is the number of initial fruits [67].

### 2.7. Ethics statement

The research was conducted on postharvest plant products obtained from natural and commercial sources and did not involve human participants, animals, or personal data. Ethical approval and informed consent were not required for this type of study.

### 2.8. Statistical analysis

All statistical analyses of the collected data were conducted using the one-way analysis (ANOVA) approach. The multiple range test [68] was used to compare means at the P ≤ 0.05 level. The data was statistically examined using the SAS program’s variance analysis function

## 3. Results

### 3.1. Physical characteristics

**Fig 2:**
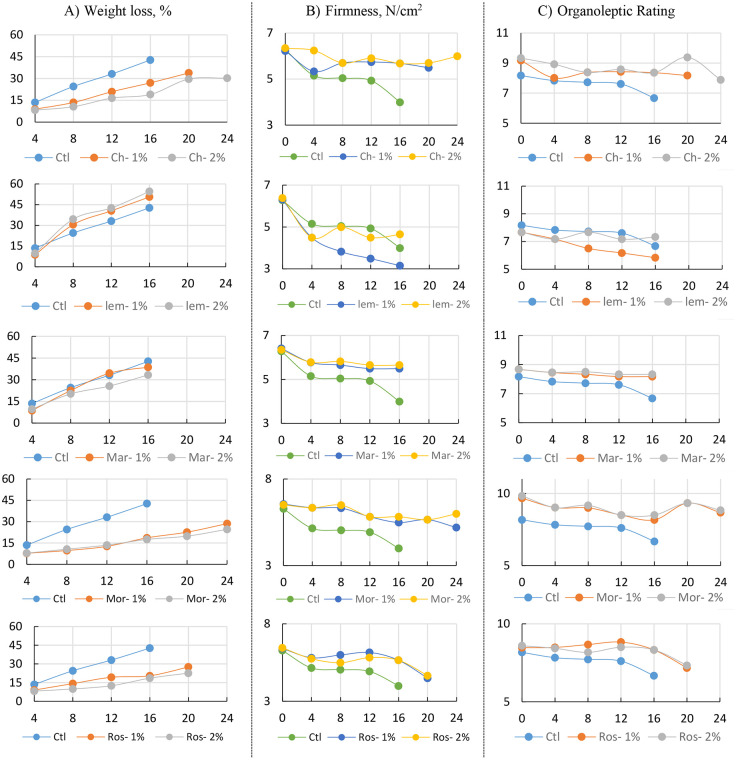
Physical Characteristics: A) weight loss %; B) firmness (N/cm^2^); and C) organoleptic rating of Guava fruits (‘Etmany’ cv.) during cold storage (8 ± 1°C, 90 ± 5% RH) as influenced by different edible coating postharvest treatments: Distilled water (Ctl), Chitosan 1% (Ch-1%), Chitosan 2% (Ch-2%), Moringa oil 1% (Mor-1%), Moringa oil 2% (Mor-2%), lemongrass oil 1% (Lem-1%), lemongrass oil 2% (Lem-2%), Marjoram 1% (Mar-1%), Marjoram 2% (Mar-2%), Rosemary 1% (Ros-1%), Rosemary 2% (Ros-2%) through the stored period: 0, 4, 8, 12, 16, 20, 24 days. Data for each parameter were displayed vertically, with five charts showing the comparison between each biodegradable material and the control.

The coatings applied to “Etmany” winter guava fruits in this study had a clear impact on their physical attributes, including weight loss percentage, firmness (N/cm²), and sensory quality. While chitosan and moringa oil showed beneficial effects, lemongrass oil and marjoram treatments had unfavorable outcomes, as detailed in supplementary data (S1–S3 Tables). Fruits treated with 2% chitosan or 1–2% moringa oil maintained quality for up to 24 days in cold storage. Similarly, those treated with 1% chitosan or 1–2% rosemary oil lasted 20 days, compared to just 16 days for untreated fruits. However, treatments with 1–2% lemongrass or marjoram oil did not extend the storage period, which remained at 16 days. Overall, the study observed a steady increase in weight loss with increasing storage duration (Fig 2).

The firmness of guava fruits showed a steady decline during storage, though the rate of softening varied depending on the treatment. The control group softened quickly, becoming less firm and unacceptable after only 12–16 days. In contrast, fruits coated with chitosan, especially at 2%, retained significantly higher firmness throughout the storage period. This suggests that chitosan helped maintain the strength of the cell walls and slowed down the softening process. Essential oils also helped preserve firmness to some extent, with 2% moringa and rosemary oils performing best, though they were not as effective as chitosan.

The least weight loss percentages were achieved by applying moringa oil (1%) for the first 12 days after storage, while applying moringa oil (2%) yielded the lowest values after 16, 20, and 24 days of storage. Regarding firmness (N/cm^2^), the highest values were observed with chitosan (2%) and moringa oil (1–2%). Generally, coating materials were effective for the organoleptic properties of fruits. Lemongrass and marjoram oils caused an adverse change in the smell and colour of guava fruits. Whereas, the fruits appeared to have a good and bright appearance when chitosan (1–2%) and moringa (1–2%) were used as edible coating materials, and the smell, colour, and taste of the fruits were not affected. The highest organoleptic values resulted when the fruits were treated with 2% chitosan and 1,2% moringa oil throughout the storage period (S1–S3 Tables).

### 3.2. Chemical characteristics

**Fig 3:**
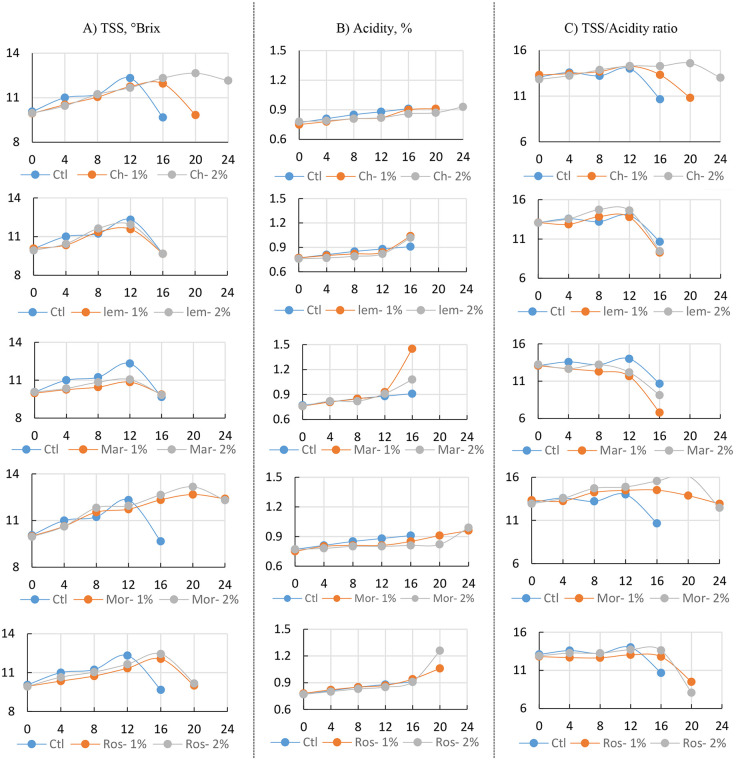
Chemical Characteristics: A) TSS; B) Acidity; and C) TSS/Acidity of Guava fruits (‘Etmany’ cv.) during cold storage (8 ± 1°C, 90 ± 5% RH) as influenced by different edible coating postharvest treatments: Distilled water (Ctl), Chitosan 1% (Ch-1%), Chitosan 2% (Ch-2%), Moringa oil 1% (Mor-1%), Moringa oil 2% (Mor-2%), lemongrass oil 1% (Lem-1%), lemongrass oil 2% (Lem-2%), Marjoram 1% (Mar-1%), Marjoram 2% (Mar-2%), Rosemary 1% (Ros-1%), Rosemary 2% (Ros-2%) through the stored period: 0, 4, 8, 12, 16, 20, 24 days. Data for each parameter were displayed vertically, with five charts showing the comparison between each biodegradable material and the control.

Our research found that coating guavas with chitosan or moringa oil significantly improved their chemical quality during cold storage. In the first few days, the untreated fruits scored highest for most chemical properties like total soluble solids (TSS), acidity, and sugars. However, this advantage faded quickly. The results indicated that total soluble solids (TSS, °Brix) increased during the early stages of storage across all treatments, reflecting the natural ripening of guava fruits. However, the extent and duration of this increase varied. Fruits in the control group reached their maximum TSS of 12.32 °Brix by day 12, followed by a sharp decline, and their quality deteriorated beyond day 16. In contrast, fruits treated with edible coatings maintained higher TSS values for a more extended period. The most notable effect was observed with 2% moringa oil, where TSS peaked at 13.17 °Brix on day 20 and remained above 12 °Brix by day 24. Moringa oil at 1% also showed strong results, sustaining 12.40 °Brix at day 24. Chitosan at 2% consistently slowed the decline, keeping TSS above 12 °Brix until the end of storage (Fig 3). Other treatments, such as lemongrass, marjoram, and rosemary, were moderately effective, but marjoram, especially at 1% and 2%, showed earlier sugar breakdown, resulting in lower final readings. Overall, higher concentrations of moringa oil and chitosan proved most effective in preserving soluble sugars and delaying fruit senescence.

Acidity patterns also differed across treatments (Fig 3B). In untreated fruits, acidity gradually increased to 0.91% by day 16, after which the fruits were no longer suitable for analysis. Chitosan treatments provided a more stable acidity profile, ranging from 0.78% to 0.93%, thereby slowing acid breakdown during ripening. Moringa oil at 2% initially showed lower acidity values (0.80% at day 8 and 0.81% at day 16), but by day 24, acidity levels rose to 0.99%, suggesting delayed yet sustained retention. A similar pattern was observed with 1% moringa oil, which maintained 0.96% by day 24. Essential oils such as marjoram, rosemary, and lemongrass gave variable results. For example, 1% marjoram resulted in the highest acidity (1.45%) during storage, which could negatively affect fruit flavor. These findings highlight that chitosan and moringa oil were most effective at maintaining balanced acidity and supporting flavor stability during extended storage.

The ratio of TSS to acidity (Fig 3C) provided an integrated measure of sweetness and flavor balance. In control fruits, this ratio peaked early at 14.01 but dropped quickly to 10.66 by day 16, reflecting over-ripening and rapid quality loss. Chitosan-coated fruits, especially at 2%, consistently maintained higher, more stable ratios, reaching 14.60 at mid-storage and remaining at 13.02 by day 24. Moringa oil at 2% achieved the best results, with the ratio peaking at 16.14, the highest among all treatments, before stabilizing at 12.47 by the end of storage. Moringa oil at 1% also performed well, maintaining a ratio of 12.92 at day 24 (S4-S6 Tables).

In contrast, marjoram led to poor outcomes, with the ratio dropping to 6.81, indicating poor sweetness and flavor balance. Lemongrass and rosemary produced intermediate results but were less effective than chitosan and moringa oil. The findings show that 2% moringa oil and 2% chitosan coatings were the most effective in maintaining sugars, balancing acidity, and preserving a favorable sweetness-to-acidity ratio. These treatments helped keep the fruits sweeter, more flavorful, and of better quality for longer.

**Fig 4:**
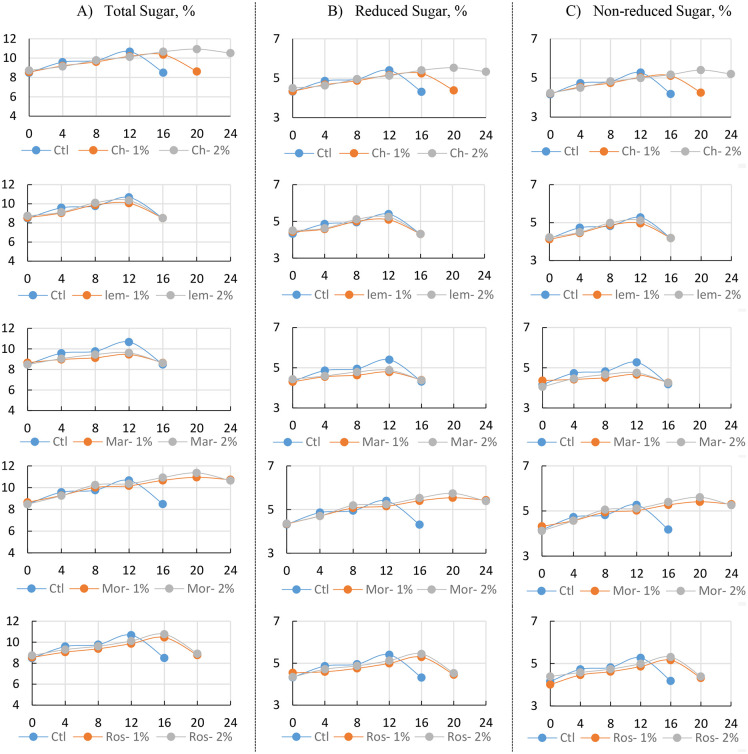
Chemical Characteristics: A) Total Sugar; B) Reduced sugar; and C) Non-reduced sugar of Guava fruits (‘Etmany’ cv.) during cold storage (8 ± 1°C, 90 ± 5% RH) as influenced by different edible coating postharvest treatments: Distilled water (Ctl), Chitosan 1% (Ch-1%), Chitosan 2% (Ch-2%), Moringa oil 1% (Mor-1%), Moringa oil 2% (Mor-2%), lemongrass oil 1% (Lem-1%), lemongrass oil 2% (Lem-2%), Marjoram 1% (Mar-1%), Marjoram 2% (Mar-2%), Rosemary 1% (Ros-1%), Rosemary 2% (Ros-2%) through the stored period: 0, 4, 8, 12, 16, 20, 24 days. Data for each parameter were displayed vertically, with five charts showing the comparison between each biodegradable material and the control.

The results showed that total sugars increased gradually in all treatments during the early stages of storage, reflecting the breakdown of starch and polysaccharides into soluble sugars as the guavas ripened. In untreated fruits, sugar levels peaked at 10.67% by day 12 but dropped quickly to 8.49% by day 16, after which the fruits deteriorated. In contrast, chitosan and essential oil coatings slowed this decline and helped sustain sugar accumulation for longer. Among them, 2% moringa oil performed best, with sugars rising steadily to 11.36% by day 20 and remaining high (10.65%) by day 24. Moringa oil at 1% and chitosan at 2% also maintained strong sugar levels, while lemongrass and marjoram were less effective, showing early declines and poor fruit quality beyond day 16. Reduced sugars, the main contributors to guava sweetness, followed a similar trend. In control fruits, levels increased early but then dropped from 5.40% on day 12 to 4.31% by day 16. Coated fruits retained higher sweetness for longer, with 2% moringa oil again proving most effective, peaking at 5.74% by day 20 and stabilizing at 5.39% by day 24. Moringa oil at 1% and chitosan at 2% also maintained high reduced sugar levels, while lemongrass and marjoram showed earlier declines. Rosemary oil provided moderate sugar retention, with 2% slightly better than 1% (Fig 4).

The same pattern was observed in non-reduced sugars (Fig 4C). Control fruits peaked at 5.27% by day 12 but declined sharply to 4.18% by day 16. With 2% moringa oil, non-reduced sugars reached 5.61% on day 20 and remained high (5.26%) even on day 24. Treatments with 1% moringa oil and 2% chitosan also effectively preserved sugar content, while marjoram and lemongrass showed poor performance, losing sugar quickly. Rosemary provided intermediate results, with higher concentrations offering slightly better retention. Overall, the findings clearly show that 2% moringa oil and 2% chitosan were the most effective coatings at preserving total, reduced, and non-reduced sugar content throughout storage. These treatments slowed down ripening-related sugar loss, maintained sweetness, and significantly extended the shelf life of winter guava compared with untreated fruits and other essential oils (S7-S9 Tables).

### 3.3. Oxidative stress markers

**Fig 5:**
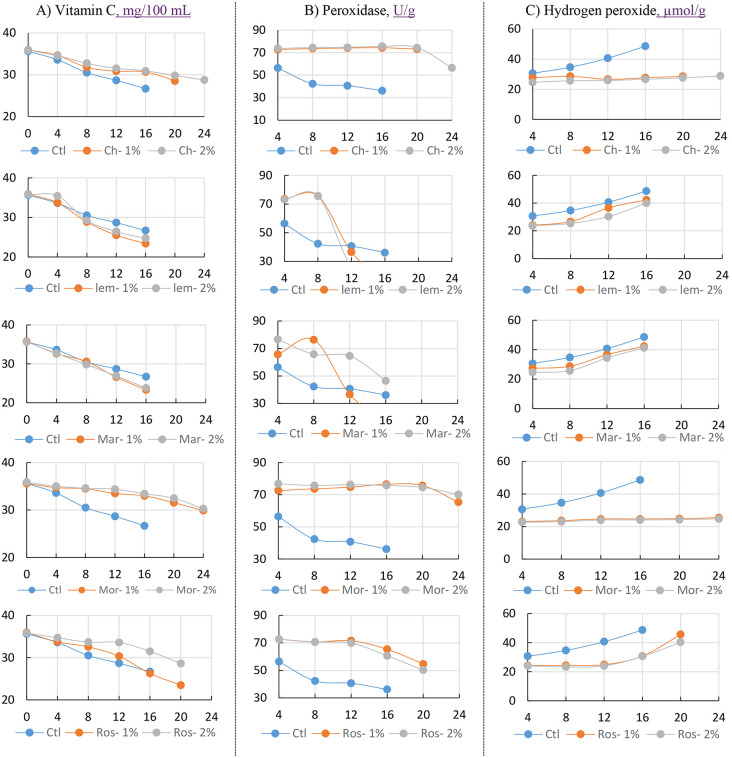
Oxidative stress markers: A) Vitamin C; B) Peroxidase; and C) Hydrogen peroxide of Guava fruits (‘Etmany’ cv.) during cold storage (8 ± 1°C, 90 ± 5% RH) as influenced by different edible coating postharvest treatments: Distilled water (Ctl), Chitosan 1% (Ch-1%), Chitosan 2% (Ch-2%), Moringa oil 1% (Mor-1%), Moringa oil 2% (Mor-2%), lemongrass oil 1% (Lem-1%), lemongrass oil 2% (Lem-2%), Marjoram 1% (Mar-1%), Marjoram 2% (Mar-2%), Rosemary 1% (Ros-1%), Rosemary 2% (Ros-2%) through the stored period: 0, 4, 8, 12, 16, 20, 24 days. Data for each parameter were displayed vertically, with five charts showing the comparison between each biodegradable material and the control.

Vitamin C (ascorbic acid) is a powerful antioxidant that protects plant tissues from oxidative stress. Guava is naturally rich in Vitamin C, which directly neutralizes reactive oxygen species (ROS) and reduces oxidative damage. Vitamin C levels in guava declined steadily during storage under all treatments, but the rate of loss varied significantly. In untreated fruits, vitamin C declined the most, from 13.5 mg/100 g at day 4 to just 42.7 mg/100 g by day 16, indicating their high vulnerability to oxidative stress and enzymatic breakdown (Fig 5).

Chitosan coatings helped slow this decline. With 1% chitosan, vitamin C decreased gradually from 48.9 mg/100 g at day 4 to 33.8 mg/100 g at day 20. The 2% treatment performed even better, dropping from 48.2 mg/100 g to 29.9 mg/100 g over the same period, suggesting that the thicker coating provided stronger protection against oxidative loss.

Among the essential oils, moringa oil provided the greatest preservation. At 1%, vitamin C declined from 48.6 mg/100 g at day 4 to 28.7 mg/100 g at day 20, while the 2% treatment retained even more, falling from 47.8 to 26.5 mg/100 g. Rosemary oil also performed well, with the 2% concentration maintaining vitamin C from 47.4 mg/100 g at day 4 to 25.3 mg/100 g at day 20, showing considerably better protection than the untreated control. By comparison, lemongrass and marjoram oils were less effective. For instance, 2% lemongrass declined from 48.0 to 22.1 mg/100 g, while 2% marjoram fell from 48.3 to 21.5 mg/100 g between day 4 and day 20. These reductions were closer to those of the control, suggesting weaker antioxidant properties than those of moringa and rosemary oils.

The results show that chitosan and moringa oil help protect guavas from oxidative stress during cold storage. This was evident from changes in peroxidase (POD) enzyme activity and hydrogen peroxide (H₂O₂) levels. Guavas treated with 2% or 1% moringa oil or 2% chitosan consistently maintained higher POD activity throughout storage. By day 16, the highest readings were 76.5 U/g for 2% moringa oil, 75.7 U/g for 1% moringa oil, and 75.7 U/g for 2% chitosan, more than double the control group, which dropped to just 36.2 U/g (Fig 5B). This higher POD activity reflects a stronger antioxidant defense, helping neutralize harmful reactive oxygen species (ROS) and preventing cell damage after harvest.

The H₂O₂ measurements further support this. In untreated fruits, H₂O₂ levels steadily rose to 48.65 µmol g ⁻ ¹ FW by day 16, signalling significant oxidative damage (Fig 5C). In contrast, fruits treated with 2% moringa oil kept H₂O₂ levels much lower, 24.3 µmol g ⁻ ¹ FW at day 20, closely followed by 1% moringa oil (24.9 µmol g ⁻ ¹ FW) and 2% chitosan (27.6 µmol g ⁻ ¹ FW). This drop in H₂O₂ aligns perfectly with the higher POD activity in these treatments, showing how effectively they limit oxidative damage and protect cell structure. Other essential oils, such as lemongrass, marjoram, and rosemary, were far less effective. Lemongrass oil, for example, showed a sharp drop in POD activity after day 12 (down to 16.67 U/g) and higher H₂O₂ levels (40.00–42.32 µmol g ⁻ ¹ FW), almost as severe as in the control. These results suggest such treatments fail to maintain a strong antioxidant defense, leading to faster deterioration. Moringa oil and chitosan were the most effective natural preservatives. Keeping POD activity high and H₂O₂ levels low slowed oxidative damage, helping guavas stay fresh, nutritious, and market-ready for much longer in cold storage (S10-S12 Tables).

### 3.4. Postharvest disease spoilage

The data in Table 1 showed continuous rises in decay due to the postharvest disease percentage (F.P.D. %) with a prolonged storage period in all studied treatments except those of chitosan 2% and moringa (1%−2%). Generally, postharvest diseases emerged in the control and marjoram oil (1–2%) treatments of fruits after 12 days of storage.

**Table 1:** Impact of chitosan and essential oils on fruit postharvest disease percentage (F.P.D. %) during cold storage conditions (at 8 ± 1°C and 90% RH) of winter guava fruits of ‘Etmany’ *cv.*

treatment	Days after Storage					
	4	8	12	16	20	24
control	0	0	15^a^	30^a^	–	–
chitosan 1%	0	0	0	0	5^b^	–
chitosan 2%	0	0	0	0	0	10^a^
lemongrass oil 1%	0	0	0	0	–	–
lemongrass oil 2%	0	0	0	0	–	–
Marjoram 1%	0	0	10^b^	25^ab^	**–**	**–**
Marjoram 2%	0	0	5^c^	20^bc^	**–**	**–**
Moringa oil 1%	0	0	0	0	0	0
Moringa oil 2%	0	0	0	0	0	0
Rosemary 1%	0	0	0	15^c^	15^a^	–
Rosemary 2%	0	0	0	10^c^	10^ab^	**–**

Means that do not share the same letters in each column are statistically different at p ≤ 0.05.

Moringa treatments (1–2%) yielded the highest results, with the least decay in guava fruits and the highest values of physiological parameters. This was followed by chitosan 2% and lemongrass oil (1–2%), with both treatments showing fewer physiological characteristics than those with moringa oil. Conversely, control and marjoram oil (1–2%) showed the highest postharvest disease percentage, followed by rosemary grass oil treatments.

## 4. Discussion

Around 30–40% of horticultural crops are subject to postharvest spoilage. Rapid changes occur due to the high rate of respiration and, consequently, the acceleration of biological processes in the fruit, reducing its quality and lowering its market value [69,70]. Guava is a climacteric fruit and therefore has a short postharvest shelf life. Guava is a subtropical fruit that is susceptible to cold damage during storage, mechanical damage, and various postharvest pathogens, which reduces its refrigerated cold storage period [71]. Using edible coatings can help keep fruits and vegetables fresher for longer by reducing water loss and respiration, thus preserving their nutritional value [72,73].

Fruit tissues lose moisture, resulting in a decrease in weight. Lowers quality, lowers market value, and causes physiological changes as well. Innovative modified coating techniques and edible coatings were used to minimise water loss. Guava coating treatments reduced weight loss, most likely by maintaining the fruit’s surface at its highest moisture content [74]. Edible coatings minimize transpiration and water loss by slowing the diffusion of water vapor from fruit. They also control the exchange of gases, such as carbon dioxide, oxygen, and water vapor, which helps maintain ideal humidity levels and prevents excessive moisture loss. Similarly, guava coated with mustard oil coating had a lower Physiological Loss in Weight (PLW) than guava without coating, most likely because the fruit’s surface retained its highest moisture content [47]. Weight reduction for Cucumber fruits progressed more slowly when coated with moringa oil-beeswax, suggesting that coating slowed weight loss [75]. In the same trend, chitosan coatings successfully stop plum fruits from losing water retarding weight loss by about 52%. These natural polysaccharide coatings help prolong the shelf life of fruits by reducing dehydration, delaying maturity, and delaying senescence [45].

Fruit firmness decreased gradually throughout storage. Enzymes soften cell walls by modifying polysaccharides, leading to solubilization, depolymerization, and the release of sugars and galacturonic acid, thereby altering cell turgidity [76]. It has been discovered that chitosan coatings reduce plum fruit firmness loss, preserving it by up to 78% compared to untreated fruits [45]. Because the chitosan coating slows down gas exchange around the fruit surface, creating a microenvironment with reduced O_2_ and accumulated CO_2_, which inhibits the activity of enzymes that soften cells. It may limit the breakdown of insoluble protopectin into more soluble pectic acid and pectin, hence preserving the overall firmness and significantly enhancing firmness retention of coated fruits [77,78]. Moringa oil as an edible coating can enhance fruit quality by maintaining firmness and minimizing mass loss while being cold stored [79]. The results of fruit hardness varied depending on the type of oil used as a coating. The firmness of mandarin coated by the Daisy oil gradually decreased during storage; mandarin coated with coconut oil maintained the highest firmness among the treatments. [80]. These results are consistent with those of [81], who found that mandarin fruits coated with coconut oil stayed solid by lowering water loss, delaying senescence, and preventing microbial activity that breaks down cell walls. Edible oils help maintain the firmness of fruit.

Chitosan and moringa oil coatings may improve the organoleptic rating of fruits by preserving their aesthetic appeal and slowing ripening [82,83]. Moringa oil’s nutritional content and flavor may give it desirable organoleptic qualities [84]. A study on sweet oranges showed that a 1% edible oil coating improved visual quality, with moringa seed oil leading to more visually appealing results, though oil type and cultivar did not significantly affect fruit appearance.

Moringa oil contains high levels of phenolic compounds, flavonoids (e.g., quercetin, kaempferol), tocopherols, sterols, and unsaturated fatty acids such as oleic acid [85]. These classes of compounds are well documented to possess strong antioxidant capacity, enabling them to scavenge ROS, including peroxides and H₂O₂ directly, and to upregulate endogenous antioxidant defenses. Where flavonoids and phenolic acids act as hydrogen donors that can neutralize free radicals, while tocopherols interrupt lipid peroxidation chain reactions by donating electrons to lipid radicals [86], this dual action lowers overall oxidative load and stabilizes cellular membranes. Furthermore, Moringa seed oil exhibits antimicrobial effects, likely linked to terpenoids, steroids, and phenolic compounds, which disrupt microbial membrane integrity and metabolic functions, thereby reducing the presence of decay and spoilage organisms on fruit surfaces [87].

Postharvest storage can lead to a temporary increase in TSS due to the breakdown of insoluble polysaccharides into sugars, which increases sucrose content [88]. The observed drop in TSS concentration follows this pattern, caused by the consumption, breakdown, and depletion of sugar during fruit respiration and ripening [47]. Early in the experiment, 2% chitosan and 1–2% moringa oil treatments likely reduced metabolic activity, thereby preserving fruit content and increasing soluble solids in later stages. The decrease in TSS content observed in the control and some treatments is attributed to the depletion, degradation, and consumption of sugars during ripening and respiration, particularly towards the end of storage [89]. The results were consistent with a study on mango and tangerine fruits coated with coconut oil [80,90] and with *Moringa oleifera*-coated Tommy Atkins mango fruits [91]. Regarding the fruit’s acidity, the coatings helped reduce it. This may be due to slowing the fruit’s respiration rate, which reduces the breakdown and decomposition of organic compounds, thereby reducing the accumulation of organic acids [92].

The edible coating significantly increased POD activity, which may be due to its ability to reduce respiration and ethylene production rates. The increased POD activity may help to extend the shelf life of the fruits by preventing the degradation of cell walls and other tissues [58]. Chitosan coating for strawberries increases catalase (CAT), polyphenol oxidase (PPO), and guaiacol peroxidase (G-POD) levels [93]. Increasing cellular H₂O₂ levels accelerates cell ageing, primarily through lipid oxidation and reactive oxygen species. H₂O₂ levels were effectively reduced in coated fruits due to increased POD enzyme activity [11,94].

Coating guava fruits with oils reduces the percentage of decay and postharvest disease compared to uncoated fruits [95]. Researchers discovered that eucalyptus and clove oils, when used as an effective coating on fruits, reduced respiration rates, weight loss, and decay rates while maintaining the overall quality of guava fruits after they were stored in cold storage for 24 days. By preventing ethylene’s effects and reducing gas exchange through the fruit skin, an edible covering can enhance protection against postharvest fruit degradation. Antimicrobial chemicals found in therapeutic plant oils, such as those from moringa, lemongrass, and rosemary, may generally limit fungal growth and delay disease onset [96].

## 5. Conclusions

This study investigated extending the cold storage of ‘Etmany’ guava (*Psidium guajava* L.) using edible coatings of chitosan or essential oils (moringa, lemongrass, marjoram, and rosemary). Results showed that a 2% chitosan solution or 1–2% moringa oil treatment prolonged cold storage viability up to 24 days, minimizing weight loss without affecting fruit quality. Moringa oil notably improved the fruit’s physiological and chemical properties, inhibited microbial growth, and reduced oxidative stress by lowering hydrogen peroxide (H₂O₂) levels through increased peroxidase (POD) activity. This research highlights the potential of natural coatings as a sustainable approach to minimizing postharvest losses in perishable fruits.

## Supporting information

S1 Table Impact of chitosan and essential oils on weight loss % during cold storage conditions (at 8 ± 1°C and 90% RH) of winter guava fruits of ‘Etmany’ cv. (DOCX)

S2 Table Impact of chitosan and essential oils on firmness (N/cm2) during cold storage conditions (at 8 ± 1°C and 90 ± 5% RH) of winter guava fruit ‘Etmany’ cv. (DOCX)

S3 Table Impact of chitosan and essential oils on organoleptic rating during cold storage conditions (at 8 ± 1°C and 90 ± 5% RH) of winter guava fruit ‘Etmany’ cv. (DOCX)

S4 Table Impact of chitosan and essential oils on total soluble solids (°Brix) during cold storage conditions (at 8 ± 1°C and 90 ± 5% RH) of winter guava fruit ‘Etmany’ cv. (DOCX)

S5 Table Impact of chitosan and essential oils on titratable acidity (%) during cold storage conditions (at 8 ± 1°C and 90 ± 5% RH) of winter guava fruit ‘Etmany’ cv. (DOCX)

S6 Table Impact of chitosan and essential oils on TSS/acidity ratio during cold storage conditions (at 8 ± 1°C and 90 ± 5% RH) of winter guava fruit ‘Etmany’ cv. (DOCX)

S7 Table Impact of chitosan and essential oils on ascorbic acid (mg/100 mL of the juice) during cold storage conditions (at 8 ± 1°C and 90 ± 5% RH) of winter guava fruit ‘Etmany’ cv. (DOCX)

S8 Table Impact of chitosan and essential oils on total sugars (%) during cold storage conditions (at 8 ± 1°C and 90 ± 5% RH) of winter guava fruit ‘Etmany’ cv. (DOCX)

S9 Table Impact of chitosan and essential oils on reduced sugars (%) during cold storage conditions (at 8 ± 1°C and 90 ± 5% RH) of winter guava fruit ‘Etmany’ cv. (DOCX)

S10 Table Impact of chitosan and essential oils on non-reduced sugars (%) during cold storage conditions (at 8 ± 1°C and 90 ± 5% RH) of winter guava fruit ‘Etmany’ cv. (DOCX)

S11 Table Impact of chitosan and essential oils on peroxidase (POD) enzyme activity (U/g) of fruits during cold storage conditions (at 8 ± 1°C and 90 ± 5% RH) of winter guava fruit ‘Etmany’ cv. (DOCX)

S12 Table Impact of chitosan and essential oils on H2O2 content (µmol g-1 FW) of fruits during cold storage conditions (at 8 ± 1°C and 90 ± 5% RH) of winter guava fruit ‘Etmany’ cv. (DOCX)

## References

[1] Abdelkader M, Suliman AA, Salem SS, Assiya A, Voronina L, Puchkov M, et al. Studying the combined impact of salinity and drought stress-simulated conditions on physio-biochemical characteristics of lettuce plant. Horticulturae. 2024;10(11):1186. doi: 10.3390/horticulturae10111186

[2] Abdelkader M, Elkhawaga FA, Suliman AA, Puchkov M, Kuranova KN, Mahmoud MH, et al. Understanding the Regular Biological Mechanism of Susceptibility of Tomato Plants to Low Incidences of Blossom-End Rot. Horticulturae. 2024;10(6):648. doi: 10.3390/horticulturae10060648

[3] Abd El-Samie KA, Khalifa EA. Impact of the present land use and environmental conditions on agricultural development at Wadi Sannur, Beni Suef, Egypt. Alexandria Science Exchange Journal. 2011;32:205–14.

[4] Kumari P, Mankar A, Karuna K, Homa F, Meiramkulova K, Siddiqui MW. Mineral composition, pigments, and postharvest quality of guava cultivars commercially grown in India. Journal of Agriculture and Food Research. 2020;2:100061. doi: 10.1016/j.jafr.2020.100061

[5] Vijaya Anand A, Velayuthaprabhu S, Rengarajan RL, Sampathkumar P, Radhakrishnan R. Bioactive Compounds of Guava (Psidium guajava L.). In: Murthy HN, Bapat VA, editors. Bioactive Compounds in Underutilized Fruits and Nuts. Cham: Springer International Publishing; 2020. 503–27. doi: 10.1007/978-3-030-30182-8_37

[6] Kumar M, Tomar M, Amarowicz R, Saurabh V, Nair MS, Maheshwari C. Guava (Psidium guajava L.) leaves: nutritional composition, phytochemical profile, and health-promoting bioactivities. Foods. 2021;10:752. doi: 10.3390/foods10040752 33916183 PMC8066327

[7] Shukla S, Kushwaha R, Singh M, Saroj R, Puranik V, Agarwal R, et al. Quantification of bioactive compounds in guava at different ripening stages. Food Res. 2021;5(3):183–9. doi: 10.26656/fr.2017.5(3).554

[8] Mohd Israfi NA, Mohd Ali MIA, Manickam S, Sun X, Goh BH, Tang SY, et al. Essential oils and plant extracts for tropical fruits protection: From farm to table. Front Plant Sci. 2022;13:999270. doi: 10.3389/fpls.2022.999270 36247633 PMC9559231

[9] Sanjay P, Saxena D, Kazimi R. Enhancing shelf life of fresh fruits by the application of different edible coatings. Int J Innov Sci. 2022;11:626–32.

[10] Thakur M, Tiwari S, Kataria S, Anand A. Recent advances in seed priming strategies for enhancing planting value of vegetable seeds. Scientia Horticulturae. 2022;305:111355. doi: 10.1016/j.scienta.2022.111355

[11] Nasser MA, El-Mogy MM, Samaan MSF, Hassan KM, El-Sayed SM, Alsubeie MS, et al. Postharvest exogenous melatonin treatment of table grape berry enhances quality and maintains bioactive compounds during refrigerated storage. Horticulturae. 2022;8(10):860. doi: 10.3390/horticulturae8100860

[12] Ali MAA, Nasser MA, Abdelhamid AN, Ali IAA, Saudy HS, Hassan KM. Melatonin as a key factor for regulating and relieving abiotic stresses in harmony with phytohormones in horticultural plants — a review. J Soil Sci Plant Nutr. 2023;24(1):54–73. doi: 10.1007/s42729-023-01586-9

[13] Alba-Jiménez JE, Vázquez-Barrios ME, Rivera-Pastrana DM, Escalona V, Mercado-Silva E. Effect of postharvest calcium treatments on firmness of guava fruit. Acta Hortic. 2018;(1194):823–30. doi: 10.17660/actahortic.2018.1194.116

[14] Abou El-Nasr MK, Hassan KM, Abd-Elhalim BT, Kucher DE, Rebouh NY, Ansabayeva A. The emerging roles of nanoparticles in managing the environmental stressors in horticulture crops—A review. Plants. 2025;14:2192. doi: 10.3390/plants14142192 40733428 PMC12298009

[15] Ding P, Lee YL. Use of essential oils for prolonging postharvest life of fresh fruits and vegetables. Int Food Res J. 2019;26:363–6.

[16] Taghavi T, Kim C, Rahemi A. Role of natural volatiles and essential oils in extending shelf life and controlling postharvest microorganisms of small fruits. Microorganisms. 2018;6(4):104. doi: 10.3390/microorganisms6040104 30301143 PMC6313609

[17] Bolouri P, Salami R, Kouhi S, Kordi M, Asgari Lajayer B, Hadian J, et al. Applications of essential oils and plant extracts in different industries. Molecules. 2022;27(24):8999. doi: 10.3390/molecules27248999 36558132 PMC9781695

[18] Hyldgaard M, Mygind T, Meyer RL. Essential oils in food preservation: mode of action, synergies, and interactions with food matrix components. Front Microbiol. 2012;3:12. doi: 10.3389/fmicb.2012.00012 22291693 PMC3265747

[19] Chouhan S, Sharma K, Guleria S. Antimicrobial activity of some essential oils—present status and future perspectives. Medicines. 2017;4:58. doi: 10.3390/medicines4030058 28930272 PMC5622393

[20] Abdelaziz HTO, Seif Mohamed EM, Younis SKA, Ahmed N, Michaeel MN, Abu-Hussien SH, et al. Selenium nanoparticle loaded on PVA/chitosan biofilm synthesized from orange peels: antimicrobial and antioxidant properties for plum preservation. BMC Chem. 2025;19(1):245. doi: 10.1186/s13065-025-01608-w 40835945 PMC12366351

[21] Moghadam M, Salami M, Mohammadian M, Khodadadi M, Emam-Djomeh Z. Development of antioxidant edible films based on mung bean protein enriched with pomegranate peel. Food Hydrocolloids. 2020;104:105735. doi: 10.1016/j.foodhyd.2020.105735

[22] Abdel Aziz MS, Salama HE, Sabaa MW. Biobased alginate/castor oil edible films for active food packaging. LWT. 2018;96:455–60. doi: 10.1016/j.lwt.2018.05.049

[23] Duguma HT. Potential applications and limitations of edible coatings for maintaining tomato quality and shelf life. Int J of Food Sci Tech. 2021;57(3):1353–66. doi: 10.1111/ijfs.15407

[24] Pellegrini N, Sbarbati R, Scarino ML, Siani V, Sieri S. Position paper on vegetarian diets from the working group of the Italian society of human nutrition. 2017. 10.1016/j.numecd.2017.10.020 29174030

[25] Tiwari A, Galanis A, Soucek MD. Biobased and environmentally benign coatings. John Wiley & Sons; 2016.

[26] Banat F, Show PL, Cocoletzi HH. Mango leaf extract incorporated chitosan antioxidant film for active food packaging. Int J Biol Macromol. 2019;126:1234–43. doi: 10.1016/j.ijbiomac.2018.12.196 30584938

[27] Prasad J, Kumar N, Pratibha Jaiswal R, Yadav A, Sharma SP, et al. Biopolymer based composite packaging: A sustainable approach for fruits and vegetables preservation. Applied Food Research. 2025;5(2):101211. doi: 10.1016/j.afres.2025.101211

[28] Hashemi SMB, Kaveh S, Abedi E, Phimolsiripol Y. Polysaccharide-based edible films/coatings for the preservation of meat and fish products: emphasis on incorporation of lipid-based nanosystems loaded with bioactive compounds. Foods. 2023;12(17):3268. doi: 10.3390/foods12173268 37685201 PMC10487091

[29] Kumar N. Polysaccharide-based component and their relevance in edible film/coating: a review. NFS. 2019;49:793–823. doi: 10.1108/NFS-10-2018-0294

[30] Kumar N, Pratibha Petkoska AT, Singla M. Natural gums for fruits and vegetables preservation: A review. In: Murthy HN, editor. Gums, resins and latexes of plant origin. Cham: Springer International Publishing; 2021. 1–37. doi: 10.1007/978-3-030-76523-1_4-1

[31] Kumar N, Pratibha Prasad J, Yadav A, Upadhyay AN, et al. Recent trends in edible packaging for food applications — perspective for the future. Food Eng Rev. 2023;15(4):718–47. doi: 10.1007/s12393-023-09358-y

[32] Thamarsha AKANWMRK, Kumar N, Pratibha Mor K, Upadhyay A, Sudhani HPK, et al. A Review of functional properties and applications of legume‐based edible coatings. Legume Science. 2024;6(4). doi: 10.1002/leg3.70004

[33] Kumar N, Upadhyay A, Shukla S, Bajpai VK, Kieliszek M, Yadav A, et al. Next generation edible nanoformulations for improving post-harvest shelf-life of citrus fruits. Food Measure. 2023;18(3):1825–56. doi: 10.1007/s11694-023-02287-8

[34] Trajkovska Petkoska A, Daniloski D, Kumar N, Pratibha Broach AT. Active edible packaging: a sustainable way to deliver functional bioactive compounds and nutraceuticals. Environmental Footprints and Eco-design of Products and Processes. Springer Singapore; 2021. p. 225–64. doi: 10.1007/978-981-16-4609-6_9

[35] Kumar N, Daniloski DP, Neeraj D’Cunha NM, Naumovski N, et al. Pomegranate peel extract - A natural bioactive addition to novel active edible packaging. Food Res Int. 2022;156:111378. doi: 10.1016/j.foodres.2022.111378 35650986

[36] Maringgal B, Hashim N, Mohamed Amin Tawakkal IS, Muda Mohamed MT. Recent advance in edible coating and its effect on fresh/fresh-cut fruits quality. Trends in Food Science & Technology. 2020;96:253–67. doi: 10.1016/j.tifs.2019.12.024

[37] Sapper M, Chiralt A. Starch-based coatings for preservation of fruits and vegetables. Coatings. 2018;8(5):152. doi: 10.3390/coatings8050152

[38] Nandane AS, Dave RK, Rao TVR. Optimization of edible coating formulations for improving postharvest quality and shelf life of pear fruit using response surface methodology. J Food Sci Technol. 2017;54(1):1–8. doi: 10.1007/s13197-016-2359-9 28242897 PMC5305695

[39] El-Sayed SM, Hassan KM, Abdelhamid AN, Yousef EE, Abdellatif YMR, Abu-Hussien SH, et al. Exogenous Paclobutrazol reinforces the antioxidant and antimicrobial properties of lavender (Lavandula officinalis L.) Oil through Modulating Its Composition of Oxygenated Terpenes. Plants (Basel). 2022;11(12):1607. doi: 10.3390/plants11121607 35736758 PMC9230930

[40] Mohamed MH, Abdelhamid AN, Ali MAA, Abd-Elhalim BT, Kandeel AM, Hassan KM. Influence of exogenously applied k-carrageenan at various concentrations on plant growth, phytochemical content, macronutrients, and essential oils of Ocimum basilicum. Sci Rep. 2025;15(1):11124. doi: 10.1038/s41598-025-93479-3 40169842 PMC11962130

[41] De Pilli T. Development of a vegetable oil and egg proteins edible film to replace preservatives and primary packaging of sweet baked goods. Food Control. 2020;114:107273. doi: 10.1016/j.foodcont.2020.107273

[42] Kazemian-Bazkiaee F, Ebrahimi A, Hosseini SM, Shojaee-Aliabadi S, Farhoodi M, Rahmatzadeh B, et al. Evaluating the protective effect of edible coatings on lipid oxidation, fatty acid composition, aflatoxins levels of roasted peanut kernels. Food Measure. 2020;14(2):1025–38. doi: 10.1007/s11694-019-00352-9

[43] Dong C, Wang B, Li F, Zhong Q, Xia X, Kong B. Effects of edible chitosan coating on Harbin red sausage storage stability at room temperature. Meat Sci. 2020;159:107919. doi: 10.1016/j.meatsci.2019.107919 31472934

[44] Dhaka RK, Kumar N, Pratibha Upadhyay A. Optimization, characterization, and influence of microfluidization on almond gum‐based composite edible film. Starch Stärke. 2021;73:2000101. doi: 10.1002/star.202000101

[45] Kumar P, Sethi S, Sharma RR, Srivastav M, Varghese E. Effect of chitosan coating on postharvest life and quality of plum during storage at low temperature. Scientia Horticulturae. 2017;226:104–9. doi: 10.1016/j.scienta.2017.08.037

[46] Jaishankar P, Nishant K, Sujata PS. Shelf-life extension of guava and eggplant fruits using chitosan-sodium alginate based antimicrobial composite coating functionalized with clove-soy lecithin nanoemulsion. Plant Sci Today. 2025. doi: 10.14719/pst.7980

[47] Singh A. Edible Oil Coatings Prolong Shelf Life and Improve Quality of Guava (Psidium guajava L.). Int J Pure App Biosci. 2017;5(3):837–43. doi: 10.18782/2320-7051.4065

[48] El-Dengawy El, Niamatt-Allah M, Wanas A, Saima A. Physical and physiological effects of pre- and post-harvest treatments using calcium chloride and jojoba oil on the guava fruits storage. Journal of Plant Production. 2018;9: 649–55. doi: 10.21608/jpp.2018.36382

[49] Sebastian S, Bala KL, Humar A. Effect of essential oil coatings and storage conditions on shelf life of guava (Psidium guajava) and amla (Emblica officinalis). The Allahabad Farmer. 2018;74.

[50] Arafat I, Dapour A, Dafea M. Effect of organic and inorganic compound and plant extracts over chemical treatments for improving quality and prolonging shelflife of guava (Psidium guajava L.). Fundam Appl Agric. 2020;1. doi: 10.5455/faa.20030

[51] Selvam SP, Mitra ADPMA, Kumar MM. Postharvest Application of Moringa Gum and Cinnamon Essential Oil as Edible Herbal Coating for Extending Shelf Life and Quality of Guava Psidium Guajava. IJEAT. 2020;9(3):4098–105. doi: 10.35940/ijeat.c6528.029320

[52] Moreira E De S, Silva NMC da, Brandão MRS, Santos HC, Ferreira Tap de C. Effect of modified starch and gelatin by-product based edible coating on the postharvest quality and shelf life of guava fruits. Food Sci Technol. 2022;42. doi: 10.1590/fst.26221

[53] Agusriansyah S, Gatot P, Neti H, Agus W. Physicochemical and microbiological properties of fruits enriched by nanoparticles as an edible coating. World J Adv Res Rev. 2023;18(2):129–33. doi: 10.30574/wjarr.2023.18.2.0772

[54] Ray A, Ghosh S, Kanjilal A, Mukherjee A, Datta S. Antimicrobial properties of chitosan nanoparticles and their role in post-harvest shelf life extension. IOSR Journal of Environmental Science, Toxicology and Food Technology. 2022;16:51–60.

[55] Makhathini N, Kumar N, Fawole OA. Enhancing circular bioeconomy: Alginate-cellulose nanofibre films/coatings functionalized with encapsulated pomegranate peel extract for postharvest preservation of pomegranate arils. Int J Biol Macromol. 2025;309(Pt 2):142848. doi: 10.1016/j.ijbiomac.2025.142848 40188910

[56] Trajkovska Petkoska A, Daniloski D, Kumar N, Pratibha Broach AT. Biobased materials as a sustainable potential for edible packaging. Environmental Footprints and Eco-design of Products and Processes. Springer Singapore; 2021. 111–35. doi: 10.1007/978-981-16-4609-6_5

[57] Mercado-Silva E, Benito-Bautista P, de los Angeles Garća-Velasco M. Fruit development, harvest index and ripening changes of guavas produced in central Mexico. Postharvest Biology and Technology. 1998;13(2):143–50. doi: 10.1016/s0925-5214(98)00003-9

[58] Mohammadi M, Rastegar S, Rohani A. Enhancing Mexican lime (Citrus aurantifolia cv.) shelf life with innovative edible coatings: xanthan gum edible coating enriched with Spirulina platensis and pomegranate seed oils. BMC Plant Biol. 2024;24(1):906. doi: 10.1186/s12870-024-05606-3 39350034 PMC11440758

[59] Pereira LL, Cardoso WS, Guarçoni RC, da Fonseca AFA, Moreira TR, Caten CS. The consistency in the sensory analysis of coffees using Q-graders. Eur Food Res Technol. 2017;243(9):1545–54. doi: 10.1007/s00217-017-2863-9

[60] Abdelkader MM, Puchkov MY, Lysakov MA, Loktionova EG, Suliman AA. Effect of crezacin and humic acid on growth and physiological aspects of tomato plants (*Lycopersicon esculentum*). JAH. 2019;21(01):61–6. doi: 10.37855/jah.2019.v21i01.11

[61] Abou El-Nasr MK, Nasser MA, Ebrahim M, Samaan MSF. Alleviating biotic stress of powdery mildew in mango cv. Keitt by Sulfur nanoparticles and assessing their effect on productivity and disease severity. Sci Rep. 2025;15(1):5537. doi: 10.1038/s41598-025-88282-z 39953098 PMC11828861

[62] Mansour N, Nasser M. A Comparison of some traditional and nontraditional organic fertilizers for murcott tangerine trees production and fruit quality. Egyptian Journal of Horticulture. 2021;48(2):241–55. doi: 10.21608/ejoh.2021.80073.1176

[63] Abou El-Nasr MK, El-Hennawy HM, Samaan MSF, Salaheldin TA, Abou El-Yazied A, El-Kereamy A. Using zinc oxide nanoparticles to improve the color and berry quality of table grapes cv. crimson seedless. Plants (Basel). 2021;10(7):1285. doi: 10.3390/plants10071285 34202840 PMC8309036

[64] Polle A, Otter T, Seifert F. Apoplastic Peroxidases and Lignification in Needles of Norway Spruce (Picea abies L.). Plant Physiol. 1994;106(1):53–60. doi: 10.1104/pp.106.1.53 12232302 PMC159498

[65] Suliman AA, Elkhawaga FA, Zargar M, Bayat M, Pakina E, Abdelkader M. Boosting Resilience and Efficiency of Tomato Fields to Heat Stress Tolerance Using Cytokinin (6-Benzylaminopurine). Horticulturae. 2024;10(2):170. doi: 10.3390/horticulturae10020170

[66] Velikova V, Yordanov I, Edreva A. Oxidative stress and some antioxidant systems in acid rain-treated bean plants. Plant Science. 2000;151(1):59–66. doi: 10.1016/s0168-9452(99)00197-1

[67] Elnagar I, Fahmy MA, Abd-Alrazik AM, Sultan MZ. Effect of some postharvest treatments with edible coating materials on storability and quality of Murcott Tangor fruits during cold storage. Al-Azhar Journal of Agricultural Research. 2021;46(1):16–27. doi: 10.21608/ajar.2021.218193

[68] Komorowski M, Marshall DC, Salciccioli JD, Crutain Y. Exploratory data analysis. Secondary analysis of electronic health records. Cham: Springer International Publishing; 2016. 185–203. doi: 10.1007/978-3-319-43742-2_15 31314267

[69] Dubey N, Chitranshi S, Dwivedi SK, Sharma A. Postharvest physiology, value chain advancement, and nanotechnology in fresh-cut fruits and vegetables. Nanotechnology horizons in food process engineering. Apple Academic Press. 2023. 99–132.

[70] El-Ramady HR, Domokos-Szabolcsy É, Abdalla NA, Taha HS, Fári M. Postharvest Management of Fruits and Vegetables Storage. Sustainable Agriculture Reviews. Springer International Publishing; 2014. 65–152. doi: 10.1007/978-3-319-09132-7_2

[71] Singh SP, Pal RK. Response of climacteric-type guava (Psidium guajava L.) to postharvest treatment with 1-MCP. Postharvest Biology and Technology. 2008;47(3):307–14. doi: 10.1016/j.postharvbio.2007.08.010

[72] Formiga AS, Pereira EM, Junior JSP, Costa FB, Mattiuz B-H. Effects of edible coatings on the quality and storage of early harvested guava. Food Chemistry Advances. 2022;1:100124. doi: 10.1016/j.focha.2022.100124

[73] Verma A, Sharma TR, Bajpai D, Sharma R, Pandey SK. Effect of edible coating on shelf life and market acceptability of Guava (*Psidium guajava* L.) cv. Lucknow-49. 2023.

[74] Gidado MJ, Gunny AAN, Gopinath SCB, Ali A, Wongs-Aree C, Salleh NHM. Challenges of postharvest water loss in fruits: Mechanisms, influencing factors, and effective control strategies – A comprehensive review. Journal of Agriculture and Food Research. 2024;17:101249. doi: 10.1016/j.jafr.2024.101249

[75] Al-Rashdi S, Al-Subhi N, Al-Dairi M, Pathare PB. Effect of a moringa oil–beeswax edible coating on the shelf-life and quality of fresh cucumber. Processes. 2024;12(6):1148. doi: 10.3390/pr12061148

[76] Rosli HG, Civello PM, Martínez GA. Changes in cell wall composition of three Fragaria x ananassa cultivars with different softening rate during ripening. Plant Physiol Biochem. 2004;42(10):823–31. doi: 10.1016/j.plaphy.2004.10.002 15596102

[77] Emragi E, Kalita D, Jayanty SS. Effect of edible coating on physical and chemical properties of potato tubers under different storage conditions. LWT. 2022;153:112580. doi: 10.1016/j.lwt.2021.112580

[78] Mshora A, Gill DP, Jawandha DS, Sinha A, Singh DM. Effect of chitosan coatings on physico-chemical and enzymatic activities in mango cv Dashehari stored at low temperature. J Hortic Sci. 2022;17:381–7. doi: 10.24154/jhs.v17i2.1015

[79] Kubheka SF, Tesfay SZ, Mditshwa A, Magwaza LS. Evaluating the efficacy of edible coatings incorporated with moringa leaf extract on postharvest of ‘maluma’ avocado fruit quality and its biofungicidal effect. horts. 2020;55(4):410–5. doi: 10.21273/hortsci14391-19

[80] Kaur G. Effect of various oil coatings on the quality and shelf life of mandarin cv. daisy. Ind J Pure App Biosci. 2020;8(3):209–20. doi: 10.18782/2582-2845.8084

[81] Nasrin S, Saha S, Begum HH, Samad R. Impacts of drought stress on growth, protein, proline, pigment content and antioxidant enzyme activities in rice (*Oryza sativa* L. var. BRRI dhan-24). Dhaka Univ J Biol Sci. 2020;29(1):117–23. doi: 10.3329/dujbs.v29i1.46537

[82] Duan C, Meng X, Meng J, Khan MdIH, Dai L, Khan A, et al. Chitosan as a preservative for fruits and vegetables: a review on chemistry and antimicrobial properties. Journal of Bioresources and Bioproducts. 2019;4(1):11–21. doi: 10.21967/jbb.v4i1.189

[83] Vieira TM, Moldão-Martins M, Alves VD. Composite coatings of chitosan and alginate emulsions with olive oil to enhance postharvest quality and shelf life of fresh figs (*Ficus carica* L. cv. ’Pingo De Mel’). Foods. 2021;10(4):718. doi: 10.3390/foods10040718 33805309 PMC8065400

[84] Ugese FD, Iordye N. Moringa and sesame seed oil coatings enhanced sensory attributes of stored sweet orange fruits. Acta Hortic. 2021;(1306):263–8. doi: 10.17660/actahortic.2021.1306.33

[85] Leone A, Spada A, Battezzati A, Schiraldi A, Aristil J, Bertoli S. Moringa oleifera seeds and oil: characteristics and uses for human health. Int J Mol Sci. 2016;17(12):2141. doi: 10.3390/ijms17122141 27999405 PMC5187941

[86] Shahbaz M, Naeem H, Batool M, Imran M, Hussain M, Mujtaba A, et al. Antioxidant, anticancer, and anti-inflammatory potential of Moringa seed and Moringa seed oil: A comprehensive approach. Food Sci Nutr. 2024;12(9):6157–73. doi: 10.1002/fsn3.4312 39554357 PMC11561834

[87] Bassey K, Mabowe M. Phytoconstituent detection, antioxidant, and antimicrobial potentials of Moringa oleifera Lam. hexane extract against selected WHO ESKAPE pathogens. Horticulturae. 2025;11:869. doi: 10.3390/horticulturae11080869

[88] Islam MK, Khan MZH, Sarkar MAR, Absar N, Sarkar SK. Changes in acidity, tss, and sugar content at different storage periods of the postharvest mango (*Mangifera indica* L.) Influenced by Bavistin DF. Int J Food Sci. 2013;2013:939385. doi: 10.1155/2013/939385 26904616 PMC4745495

[89] Al Lawati R, Al Shukaili Z, Al-Dairi M, Pathare PB. Effect of aloe-vera coating on the quality of mechanically damaged zucchini during long-term storage. Sustainable Chemistry and Pharmacy. 2024;39:101603. doi: 10.1016/j.scp.2024.101603

[90] Nasrin TAA, Islam MN, Rahman MA, Arfin MS, Ullah MA. Evaluation of postharvest quality of edible coated mandarin at ambient storage. 2018. 10.22004/AG.ECON.305444

[91] Silva S, Sousa AP, Gomes J, Andrade R, Lima G, Siqueira E, et al. Postharvest conservation of Tommy atkins mango fruits during storage using Moringa oleifera oil-based coating. Aust J Crop Sci. 2021;(15(02):2021):278–83. doi: 10.21475/ajcs.21.15.02.p2984

[92] Eshetu A, Ibrahim AM, Forsido SF, Kuyu CG. Effect of beeswax and chitosan treatments on quality and shelf life of selected mango (Mangifera indica L.) cultivars. Heliyon. 2019;5:e01116. doi: 10.1016/j.heliyon.2018.e01116 PMC6328066 30671558

[93] Badawy MEI, Rabea EI, A. M. El-Nouby M, Ismail RIA, Taktak NEM. Strawberry shelf life, composition, and enzymes activity in response to edible chitosan coatings. International Journal of Fruit Science. 2016;17(2):117–36. doi: 10.1080/15538362.2016.1219290

[94] Hassan AH, Mansour N, Samaan MSF, Nasser MA. Improving naomi mango trees capability to withstand salt stress using some plant growth regulators. J Soil Sci Plant Nutr. 2025;25(3):7152–69. doi: 10.1007/s42729-025-02586-7

[95] El-Bana H, Ennab H. Effect of some natural oils on prolonging the storage period of winter Guava Fruits (*Psidium guajava* L.). Alexandria Science Exchange Journal. 2023;44(4):551–62. doi: 10.21608/asejaiqjsae.2023.319909

[96] Hassan ZH, Lesmayati S, Qomariah R, Hasbianto A. Effects of wax coating applications and storage temperatures on the quality of tangerine citrus (*Citrus reticulata*) var. Siam Banjar. International Food Research Journal. 2014;21.

