# Supplementary material for: Sustainable chitosan and medicinal plant oils as natural edible coatings for postharvest quality preservation of guava fruits (Psidium guajava L.)
Source: PLoS One. 2026 Mar 18;21(3):e0342650. doi: 10.1371/journal.pone.0342650 (PMC12998884; doi:10.1371/journal.pone.0342650)
Supplement: S1 Table — (DOCX) [file pone.0342650.s001.docx]

**S1 Table:** Impact of chitosan and essential oils on weight loss % during cold storage conditions (at 8±1°C and 90 % RH) of winter guava fruits of ‘Etmany’ *cv*.

| Treatment | Days after cold storage | | | | | |
| --- | --- | --- | --- | --- | --- | --- |
|  | 4 | 8 | 12 | 16 | 20 | 24 |
| control | 13.50±0.50^a^ | 24.50±0.50^c^ | 33.16±0.17^d^ | 42.71±0.03^c^ | - | - |
| chitosan 1% | 8.91±0.23^c^ | 13.55±0.10^g^ | 20.90±0.05^f^ | 27.01±0.05^f^ | 33.81±0.04^a^ | - |
| chitosan 2% | 8.22±0.33^de^ | 10.61±0.06^h^ | 16.55±0.10^h^ | 19.08±0.04^h^ | 29.63±0.04^b^ | 30.2±0.02^a^ |
| lemongrass oil 1% | 8.67±0.03^cd^ | 30.55±0.10^b^ | 40.55±0.10^b^ | 50.63±0.03^b^ | - | - |
| lemongrass oil 2% | 9.66±0.12^b^ | 34.52±0.23^a^ | 42.62±0.05^a^ | 54.62±0.03^a^ | - | - |
| Marjoram 1% | 8.65±0.09^cd^ | 22.59±0.05^d^ | 34.55±0.10^c^ | 38.60±0.07^d^ | - | - |
| Marjoram 2% | 9.68±0.06^b^ | 20.26±0.09^e^ | 25.60±0.06^e^ | 33.30±0.02^e^ | - | - |
| Moringa oil 1% | 7.75±0.10^e^ | 9.680±0.06^i^ | 12.62±0.07^j^ | 18.60±0.05^i^ | 22.63±0.03^d^ | 28.61±0.05^b^ |
| Moringa oil 2% | 7.85±0.10^e^ | 10.62±0.05^h^ | 13.59±0.05^i^ | 17.59±0.06^j^ | 19.81±0.03^e^ | 24.62±0.04^c^ |
| Rosemary 1% | 9.05±0.01^bc^ | 14.21±0.18^f^ | 19.31±0.02^g^ | 20.59±0.07^g^ | 27.62±0.03^c^ | - |
| Rosemary 2% | 8.19±0.27^de^ | 9.93±0.05^i^ | 12.56±0.09^j^ | 18.61±0.04^j^ | 22.61±0.03^d^ | - |

The data were presented as mean ± SD (standard deviation). According to the Tukey test, means that do not share the letters for each variable in each column differ significantly at p≤ 0.05.
