# Supplementary material for: Sustainable chitosan and medicinal plant oils as natural edible coatings for postharvest quality preservation of guava fruits (Psidium guajava L.)
Source: PLoS One. 2026 Mar 18;21(3):e0342650. doi: 10.1371/journal.pone.0342650 (PMC12998884; doi:10.1371/journal.pone.0342650)
Supplement: S2 Table — (DOCX) [file pone.0342650.s002.docx]

**S2 Table**: Impact of chitosan and essential oils on firmness (N/cm^2^) during cold storage conditions (at 8±1°C and 90±5% RH) of winter guava fruit ‘Etmany’ *cv.*

| treatment | Days after cold storage | | | | | | |
| --- | --- | --- | --- | --- | --- | --- | --- |
|  | 0 | 4 | 8 | 12 | 16 | 20 | 24 |
| control | 6.28±0.06^a^ | 5.15±0.29^ab^ | 5.04±0.26^b^ | 4.93±0.35^bc^ | 3.99±0.29^cd^ | - | - |
| chitosan 1% | 6.22±0.06^a^ | 5.33±0.87^ab^ | 5.70±0.34^ab^ | 5.74±0.38^ab^ | 5.68±0.31^ab^ | 5.49±0.29^a^ | - |
| chitosan 2% | 6.34±0.21^a^ | 6.24±0.38^a^ | 5.70±0.34^ab^ | 5.90±0.52^ab^ | 5.68±0.31^ab^ | 5.70±0.34^a^ | 5.99±0.29^a^ |
| lemongrass oil 1% | 6.31±0.20^a^ | 4.49±0.29^b^ | 3.82±0.50^c^ | 3.49±0.29^d^ | 3.15±0.76^d^ | - | - |
| lemongrass oil 2% | 6.38±0.06^a^ | 4.49±0.29^b^ | 4.99±0.29^b^ | 4.49±0.29^cd^ | 4.65±0.29^bc^ | - | - |
| Marjoram 1% | 6.41±0.15^a^ | 5.77±0.43^ab^ | 5.65±0.29^ab^ | 5.49±0.29^a-c^ | 5.49±0.29^ab^ | - | - |
| Marjoram 2% | 6.35±0.10^a^ | 5.78±0.45^ab^ | 5.82±0.50^ab^ | 5.65±0.29^ab^ | 5.65±0.29^ab^ | - | - |
| Moringa oil 1% | 6.55±0.10^a^ | 6.34±0.53^a^ | 6.32±0.50^a^ | 5.82±0.50^ab^ | 5.49±0.29^ab^ | 5.65±0.29^a^ | 5.20±0.83^a^ |
| Moringa oil 2% | 6.51±0.16^a^ | 6.34±0.53^a^ | 6.49±0.29^a^ | 5.82±0.50^ab^ | 5.82±0.50^a^ | 5.65±0.29^a^ | 5.99±0.29^a^ |
| Rosemary 1% | 6.41±0.11^a^ | 5.81±0.49^ab^ | 5.99±0.29^ab^ | 6.15±0.29^a^ | 5.65±0.29^ab^ | 4.49±0.29^c^ | - |
| Rosemary 2% | 6.45±0.10^a^ | 5.75±0.40^ab^ | 5.49±0.29^ab^ | 5.82±0.50^ab^ | 5.65±0.29^ab^ | 4.65±0.29^c^ | - |

The data were presented as mean ± SD (standard deviation). According to the Tukey test, means that do not share the letters for each variable in each column differ significantly at p≤ 0.05.
