# Supplementary material for: Sustainable chitosan and medicinal plant oils as natural edible coatings for postharvest quality preservation of guava fruits (Psidium guajava L.)
Source: PLoS One. 2026 Mar 18;21(3):e0342650. doi: 10.1371/journal.pone.0342650 (PMC12998884; doi:10.1371/journal.pone.0342650)
Supplement: S3 Table — (DOCX) [file pone.0342650.s003.docx]

**S3 Table:** Impact of chitosan and essential oils on organoleptic rating during cold storage conditions (at 8±1°C and 90±5% RH) of winter guava fruit ‘Etmany’ *cv*.

| treatment | Days after cold storage | | | | | | |
| --- | --- | --- | --- | --- | --- | --- | --- |
|  | 0 | 4 | 8 | 12 | 16 | 20 | 24 |
| control | 8.17±0.29^de^ | 7.83±0.29^ab^ | 7.72±0.26^b^ | 7.61±0.35^bc^ | 6.67±0.29^cd^ | - | - |
| chitosan 1% | 9.17±0.29^a-c^ | 8.01±0.87^ab^ | 8.38±0.34^ab^ | 8.42±0.38^ab^ | 8.36±0.31^ab^ | 8.17±0.29^b^ | - |
| chitosan 2% | 9.33±0.20^ab^ | 8.92±0.38^a^ | 8.38±0.34^ab^ | 8.58±0.52^ab^ | 8.36±0.31^ab^ | 9.38±0.34^a^ | 7.88±0.29^a^ |
| lemongrass oil 1% | 7.67±0.29^e^ | 7.17±0.29^b^ | 6.50±0.50^c^ | 6.17±0.29^d^ | 5.83±0.76^d^ | - | - |
| lemongrass oil 2% | 7.67±0.27^e^ | 7.17±0.29^b^ | 7.67±0.29^b^ | 7.17±0.29^cd^ | 7.33±0.29^bc^ | - | - |
| Marjoram 1% | 8.67±0.29^b-d^ | 8.45±0.43^ab^ | 8.33±0.29^ab^ | 8.17±0.29^a-c^ | 8.17±0.29^ab^ | - | - |
| Marjoram 2% | 8.67±0.28^b-d^ | 8.46±0.45^ab^ | 8.50±0.50^ab^ | 8.33±0.29^ab^ | 8.33±0.29^ab^ | - | - |
| Moringa oil 1% | 9.67±0.20^a^ | 9.02±0.53^a^ | 9.00±0.50^a^ | 8.50±0.50^ab^ | 8.17±0.29^ab^ | 9.33±0.29^a^ | 8.67±0.83^a^ |
| Moringa oil 2% | 9.83±0.28^a^ | 9.02±0.53^a^ | 9.17±0.29^a^ | 8.50±0.50^ab^ | 8.50±0.50^a^ | 9.33±0.29^a^ | 8.83±0.29^a^ |
| Rosemary 1% | 8.47±0.06^c-e^ | 8.49±0.49^ab^ | 8.67±0.29^ab^ | 8.83±0.29^a^ | 8.33±0.29^ab^ | 7.17±0.29^c^ | - |
| Rosemary 2% | 8.60±0.36^b-d^ | 8.43±0.40^ab^ | 8.17±0.29^ab^ | 8.50±0.50^ab^ | 8.33±0.29^ab^ | 7.33±0.29^c^ | - |

The data were presented as mean ± SD (standard deviation). According to the Tukey test, means that do not share the letters for each variable in each column differ significantly at p≤ 0.05.
