# Supplementary material for: Sustainable chitosan and medicinal plant oils as natural edible coatings for postharvest quality preservation of guava fruits (Psidium guajava L.)
Source: PLoS One. 2026 Mar 18;21(3):e0342650. doi: 10.1371/journal.pone.0342650 (PMC12998884; doi:10.1371/journal.pone.0342650)
Supplement: S4 Table — (DOCX) [file pone.0342650.s004.docx]

**S4 Table:** Impact of chitosan and essential oils on total soluble solids (°Brix) during cold storage conditions (at 8±1°C and 90±5% RH) of winter guava fruit ‘Etmany’ *cv.*

| Treatment | Days after cold storage | | | | | | |
| --- | --- | --- | --- | --- | --- | --- | --- |
|  | 0 | 4 | 8 | 12 | 16 | 20 | 24 |
| control | 10.06±0.13^a^ | 11.00±0.02^a^ | 11.23±0.03^d^ | 12.32±0.03^a^ | 9.67±0.29^b^ | - | - |
| chitosan 1% | 9.94±0.12^a^ | 10.53±0.03^bc^ | 11.04±0.03^e^ | 11.75±0.05^c^ | 11.95±0.05^a^ | 9.83±0.29^b^ | - |
| chitosan 2% | 9.96±0.08^a^ | 10.45±0.05^cd^ | 11.23±0.03^d^ | 11.67±0.03^cd^ | 12.32±0.03^a^ | 12.65±0.05^a^ | 12.15±0.13^b^ |
| lemongrass oil 1% | 10.08±0.12^a^ | 10.34±0.03^de^ | 11.35±0.05^c^ | 11.58±0.08^d^ | 9.67±0.76^b^ | - | - |
| lemongrass oil 2% | 9.93±0.11^a^ | 10.44±0.03^cd^ | 11.63±0.03^b^ | 11.94±0.04^b^ | 9.67±0.76^b^ | - | - |
| Marjoram 1% | 9.97±0.09^a^ | 10.25±0.05^e^ | 10.45±0.05^g^ | 10.85±0.05^g^ | 9.87±0.4^b^ | - | - |
| Marjoram 2% | 10.06±0.13^a^ | 10.35±0.05^de^ | 10.84±0.04^f^ | 11.05±0.05^f^ | 9.83±0.76^b^ | - | - |
| Moringa oil 1% | 10.00±0.21^a^ | 10.63±0.03^b^ | 11.54±0.03^b^ | 11.73±0.02^c^ | 12.33±0.03^a^ | 12.67±0.29^a^ | 12.4±0.05^a^ |
| Moringa oil 2% | 9.96±0.08^a^ | 10.62±0.10^b^ | 11.82±0.03^a^ | 11.95±0.05^b^ | 12.65±0.05^a^ | 13.17±0.28^a^ | 12.31±0.04^ab^ |
| Rosemary 1% | 9.94±0.12^a^ | 10.35±0.05^de^ | 10.74±0.04^f^ | 11.34±0.03^e^ | 12.06±0.07^a^ | 10.00±0.50^b^ | - |
| Rosemary 2% | 9.93±0.08^a^ | 10.65±0.05^b^ | 11.04±0.03^e^ | 11.65±0.05^cd^ | 12.44±0.03^a^ | 10.17±0.29^b^ | - |

The data were presented as mean ± SD (standard deviation). According to the Tukey test, means that do not share the letters for each variable in each column differ significantly at p≤ 0.05.
