# Supplementary material for: Sustainable chitosan and medicinal plant oils as natural edible coatings for postharvest quality preservation of guava fruits (Psidium guajava L.)
Source: PLoS One. 2026 Mar 18;21(3):e0342650. doi: 10.1371/journal.pone.0342650 (PMC12998884; doi:10.1371/journal.pone.0342650)
Supplement: S5 Table — (DOCX) [file pone.0342650.s005.docx]

**S5 Table**: Impact of chitosan and essential oils on titratable acidity (%) during cold storage conditions (at 8±1°C and 90±5% RH) of winter guava fruit ‘Etmany’ *cv*.

| treatment | Days after cold storage | | | | | | |
| --- | --- | --- | --- | --- | --- | --- | --- |
|  | 0 | 4 | 8 | 12 | 16 | 20 | 24 |
| control | 0.77±0.02^a^ | 0.81±0.01^ab^ | 0.85±0.01^a^ | 0.88±0.01^bc^ | 0.91±0.01^cd^ | - | - |
| chitosan 1% | 0.75±0.02^a^ | 0.78±0.02^cd^ | 0.81±0.01^cd^ | 0.82±0.01^e-g^ | 0.9±0.01^cd^ | 0.91±0.01^c^ | - |
| chitosan 2% | 0.78±0.01^a^ | 0.79±0.01^b-d^ | 0.81±0.01^b-d^ | 0.82±0.02^e-g^ | 0.86±0.01^d^ | 0.87±0.01^cd^ | 0.93±0.01^c^ |
| lemongrass oil 1% | 0.77±0.03 | 0.80±0.01^a-c^ | 0.82±0.01^bc^ | 0.84±0.01^d-f^ | 1.04±0.06^bc^ | - | - |
| lemongrass oil 2% | 0.76±0.04^a^ | 0.77±0.01^d^ | 0.79±0.01^d^ | 0.82±0.02^e-g^ | 1.02±0.02^bc^ | - | - |
| Marjoram 1% | 0.76±0.02^a^ | 0.81±0.01^ab^ | 0.85±0.01^a^ | 0.93±0.01^a^ | 1.45±0.11^a^ | - | - |
| Marjoram 2% | 0.76±0.01^a^ | 0.82±0.01^a^ | 0.82±0.01^bc^ | 0.91±0.02^ab^ | 1.08±0.12^b^ | - | - |
| Moringa oil 1% | 0.75±0.01^a^ | 0.80±0.01^a-c^ | 0.81±0.01^b-d^ | 0.81±0.01^fg^ | 0.85±0.01^d^ | 0.91±0.01^c^ | 0.96±0.01^b^ |
| Moringa oil 2% | 0.77±0.02^a^ | 0.78±0.01^cd^ | 0.80±0.01^cd^ | 0.8±0.01^g^ | 0.81±0.01^d^ | 0.82±0.02^d^ | 0.99±0.01^a^ |
| Rosemary 1% | 0.78±0.03^a^ | 0.82±0.01^a^ | 0.85±0.01^a^ | 0.87±0.01^cd^ | 0.94±0.01^b-d^ | 1.06±0.06^b^ | - |
| Rosemary 2% | 0.77±0.02^a^ | 0.80±0.01^a-c^ | 0.83±0.01^ab^ | 0.85±0.01^c-e^ | 0.91±0.02^cd^ | 1.26±0.05^a^ | - |

The data were presented as mean ± SD (standard deviation). According to the Tukey test, means that do not share the letters for each variable in each column differ significantly at p≤ 0.05.
