# Supplementary material for: Sustainable chitosan and medicinal plant oils as natural edible coatings for postharvest quality preservation of guava fruits (Psidium guajava L.)
Source: PLoS One. 2026 Mar 18;21(3):e0342650. doi: 10.1371/journal.pone.0342650 (PMC12998884; doi:10.1371/journal.pone.0342650)
Supplement: S6 Table — (DOCX) [file pone.0342650.s006.docx]

**S6 Table**: Impact of chitosan and essential oils on TSS/acidity ratio during cold storage conditions (at 8±1°C and 90±5% RH) of winter guava fruit ‘Etmany’ *cv*.

| treatment | Days after cold storage | | | | | | |
| --- | --- | --- | --- | --- | --- | --- | --- |
|  | 0 | 4 | 8 | 12 | 16 | 20 | 24 |
| control | 13.08±0.38^a^ | 13.58±0.16^a^ | 13.21±0.14^e^ | 14.01±0.13^c-e^ | 10.66±0.39^d^ | - | - |
| chitosan 1% | 13.31±0.14^a^ | 13.45±0.27^a^ | 13.68±0.06^cd^ | 14.27±0.12^b-e^ | 13.33±0.05^bc^ | 10.81±0.34^c^ | - |
| chitosan 2% | 12.83±0.15^a^ | 13.23±0.15^ab^ | 13.86±0.19^bc^ | 14.29±0.26^b-d^ | 14.28±0.07^ab^ | 14.6±0.15^b^ | 13.02±0.11^a^ |
| lemongrass oil 1% | 13.09±0.24^a^ | 12.87±0.07^bc^ | 13.84±0.21^bc^ | 13.79±0.08^de^ | 9.28±0.9^de^ | - | - |
| lemongrass oil 2% | 13.07±0.36^a^ | 13.55±0.22^a^ | 14.73±0.22^a^ | 14.63±0.3^ab^ | 9.46±0.95^de^ | - | - |
| Marjoram 1% | 13.08±0.62^a^ | 12.66±0.14^c^ | 12.3±0.18^f^ | 11.67±0.16^g^ | 6.81±0.46^f^ | - | - |
| Marjoram 2% | 13.24±0.33^a^ | 12.67±0.05^c^ | 13.22±0.13^de^ | 12.19±0.33^g^ | 9.14±0.75^e^ | - | - |
| Moringa oil 1% | 13.33±0.10^a^ | 13.24±0.08^ab^ | 14.24±0.21^b^ | 14.48±0.21^a-c^ | 14.51±0.14^ab^ | 13.87±0.5^b^ | 12.92±0.12^a^ |
| Moringa oil 2% | 12.94±0.27^a^ | 13.55±0.23^a^ | 14.72±0.13^a^ | 14.88±0.16^a^ | 15.56±0.23^a^ | 16.14±0.64^a^ | 12.47±0.16^b^ |
| Rosemary 1% | 12.80±0.39^a^ | 12.67±0.11^c^ | 12.64±0.1^f^ | 13.03±0.15^f^ | 12.79±0.16^bc^ | 9.49±0.87^c^ | - |
| Rosemary 2% | 12.90±0.34^a^ | 13.26±0.15^ab^ | 13.24±0.07^de^ | 13.71±0.14^e^ | 13.62±0.2^bc^ | 8.07±0.1^d^ | - |

The data were presented as mean ± SD (standard deviation). According to the Tukey test, means that do not share the letters for each variable in each column differ significantly at p≤ 0.05.
