# Supplementary material for: Sustainable chitosan and medicinal plant oils as natural edible coatings for postharvest quality preservation of guava fruits (Psidium guajava L.)
Source: PLoS One. 2026 Mar 18;21(3):e0342650. doi: 10.1371/journal.pone.0342650 (PMC12998884; doi:10.1371/journal.pone.0342650)
Supplement: S7 Table — (DOCX) [file pone.0342650.s007.docx]

**S7 Table**: Impact of chitosan and essential oils on ascorbic acid (mg/100 mL of the juice) during cold storage conditions (at 8±1°C and 90±5% RH) of winter guava fruit ‘Etmany’ *cv*.

| Treatment | Days after cold storage | | | | | | |
| --- | --- | --- | --- | --- | --- | --- | --- |
|  | 0 | 4 | 8 | 12 | 16 | 20 | 24 |
| control | 35.64±0.39^a^ | 33.60±0.05^c^ | 30.51±0.15^e^ | 28.68±0.06^e^ | 26.68±0.15^d^ | - | - |
| chitosan 1% | 35.96±0.36^a^ | 34.65±0.01^b^ | 31.78±0.12^d^ | 30.87±0.02^d^ | 30.68±0.06^c^ | 28.56±0.60^d^ | - |
| chitosan 2% | 35.89±0.21^a^ | 34.59±0.05^b^ | 32.76±0.19^c^ | 31.54±0.10^c^ | 30.93±0.06^c^ | 29.83±0.04^c^ | 28.76±0.19^b^ |
| lemongrass oil 1% | 35.89±0.21^a^ | 33.72±0.12^c^ | 28.86±0.20^g^ | 25.50±0.14^g^ | 23.38±0.34^f^ | - | - |
| lemongrass oil 2% | 35.83±0.55^a^ | 35.43±0.38^a^ | 29.22±0.50^fg^ | 26.43±0.38^f^ | 24.73±0.14^e^ | - | - |
| Marjoram 1% | 35.77±0.20^a^ | 32.62±0.05^d^ | 30.58±0.12^e^ | 26.58±0.06^f^ | 23.30±0.33^f^ | - | - |
| Marjoram 2% | 35.64±0.39^a^ | 32.69±0.27^d^ | 29.80±0.13^f^ | 26.90±0.09^f^ | 23.78±0.12^f^ | - | - |
| Moringa oil 1% | 35.60±0.32^a^ | 34.72±0.12^b^ | 34.49±0.21^a^ | 33.50±0.15^b^ | 32.98±0.08^a^ | 31.54±0.09^b^ | 29.90±0.07^a^ |
| Moringa oil 2% | 35.88±0.40^a^ | 35.01±0.04^ab^ | 34.61±0.07^a^ | 34.39±0.34^a^ | 33.43±0.02^a^ | 32.49±0.21^a^ | 30.28±0.24^a^ |
| Rosemary 1% | 35.96±0.36^a^ | 33.75±0.16^c^ | 32.56±0.01^c^ | 30.34±0.39^d^ | 26.28±0.26^d^ | 23.50±0.17^e^ | - |
| Rosemary 2% | 35.84±0.18^a^ | 34.72±0.12^b^ | 33.68±0.16^b^ | 33.60±0.01^b^ | 31.48±0.14^b^ | 28.61±0.06^d^ | - |

The data were presented as mean ± SD (standard deviation). According to the Tukey test, means that do not share the letters for each variable in each column differ significantly at p≤ 0.05.
