# Supplementary material for: Sustainable chitosan and medicinal plant oils as natural edible coatings for postharvest quality preservation of guava fruits (Psidium guajava L.)
Source: PLoS One. 2026 Mar 18;21(3):e0342650. doi: 10.1371/journal.pone.0342650 (PMC12998884; doi:10.1371/journal.pone.0342650)
Supplement: S8 Table — (DOCX) [file pone.0342650.s008.docx]

**S8 Table**: Impact of chitosan and essential oils on total sugars (%) during cold storage conditions (at 8±1°C and 90±5% RH) of winter guava fruit ‘Etmany’ *cv*.

| treatment | Days after cold storage | | | | | | |
| --- | --- | --- | --- | --- | --- | --- | --- |
|  | 0 | 4 | 8 | 12 | 16 | 20 | 24 |
| control | 8.48±0.10^a^ | 9.58±0.02^a^ | 9.76±0.02^d^ | 10.67±0.02^a^ | 8.49±0.24^b^ | - | - |
| chitosan 1% | 8.55±0.12^a^ | 9.20±0.02^bc^ | 9.61±0.03^e^ | 10.19±0.04^c^ | 10.36±0.04^a^ | 8.62±0.24^b^ | - |
| chitosan 2% | 8.73±0.04^a^ | 9.13±0.04^cd^ | 9.76±0.02^d^ | 10.12±0.02^cd^ | 10.66±0.02^a^ | 10.93±0.04^a^ | 10.52±0.11^b^ |
| lemongrass oil 1% | 8.55±0.13^a^ | 9.04±0.03^de^ | 9.87±0.04^c^ | 10.06±0.06^d^ | 8.49±0.62^b^ | - | - |
| lemongrass oil 2% | 8.73±0.04^a^ | 9.12±0.03^cd^ | 10.10±0.02^b^ | 10.35±0.03^b^ | 8.49±0.62^b^ | - | - |
| Marjoram 1% | 8.66±0.21^a^ | 8.97±0.04^e^ | 9.13±0.04^g^ | 9.46±0.04^g^ | 8.65±0.33^b^ | - | - |
| Marjoram 2% | 8.48±0.12^a^ | 9.05±0.04^de^ | 9.45±0.03^f^ | 9.62±0.04^f^ | 8.62±0.62^b^ | - | - |
| Moringa oil 1% | 8.66±0.21^a^ | 9.28±0.02^b^ | 10.02±0.03^b^ | 10.17±0.02^c^ | 10.67±0.02^a^ | 10.95±0.24^a^ | 10.73±0.04^a^ |
| Moringa oil 2% | 8.48±0.10^a^ | 9.26±0.09^b^ | 10.25±0.02^a^ | 10.36±0.04^b^ | 10.93±0.04^a^ | 11.36±0.23^a^ | 10.65±0.03^ab^ |
| Rosemary 1% | 8.55±0.14^a^ | 9.05±0.04^de^ | 9.37±0.03^f^ | 9.86±0.03^e^ | 10.45±0.05^a^ | 8.76±0.41^b^ | - |
| Rosemary 2% | 8.73±0.04^a^ | 9.29±0.04^b^ | 9.61±0.03^e^ | 10.11±0.04^cd^ | 10.76±0.03^a^ | 8.90±0.24^b^ | - |

The data were presented as mean ± SD (standard deviation). According to the Tukey test, means that do not share the letters for each variable in each column differ significantly at p≤ 0.05.
