# Supplementary material for: Sustainable chitosan and medicinal plant oils as natural edible coatings for postharvest quality preservation of guava fruits (Psidium guajava L.)
Source: PLoS One. 2026 Mar 18;21(3):e0342650. doi: 10.1371/journal.pone.0342650 (PMC12998884; doi:10.1371/journal.pone.0342650)
Supplement: S9 Table — (DOCX) [file pone.0342650.s009.docx]

**S9 Table**: Impact of chitosan and essential oils on reduced sugars (%) during cold storage conditions (at 8±1°C and 90±5% RH) of winter guava fruit ‘Etmany’ *cv*.

| treatment | Days after cold storage | | | | | | |
| --- | --- | --- | --- | --- | --- | --- | --- |
|  | 0 | 4 | 8 | 12 | 16 | 20 | 24 |
| control | 4.32±0.06^a^ | 4.86±0.01^a^ | 4.95±0.01^d^ | 5.40±0.01^a^ | 4.31±0.12^b^ | - | - |
| chitosan 1% | 4.34±0.05^a^ | 4.67±0.01^bc^ | 4.87±0.01^e^ | 5.16±0.02^c^ | 5.24±0.02^a^ | 4.38±0.12^b^ | - |
| chitosan 2% | 4.50±0.25^a^ | 4.63±0.02^cd^ | 4.95±0.01^d^ | 5.13±0.01^cd^ | 5.40±0.01^a^ | 5.53±0.02^a^ | 5.33±0.05^b^ |
| lemongrass oil 1% | 4.43±0.04^a^ | 4.58±0.01^de^ | 5.00±0.02^c^ | 5.09±0.03^d^ | 4.31±0.31^b^ | - | - |
| lemongrass oil 2% | 4.50±0.24^a^ | 4.62±0.01^cd^ | 5.11±0.01^b^ | 5.24±0.02^b^ | 4.31±0.31^b^ | - | - |
| Marjoram 1% | 4.30±0.06^a^ | 4.55±0.02^e^ | 4.63±0.02^g^ | 4.79±0.02^g^ | 4.39±0.17^b^ | - | - |
| Marjoram 2% | 4.43±0.04^a^ | 4.59±0.02^de^ | 4.79±0.01^f^ | 4.88±0.02^f^ | 4.38±0.31^b^ | - | - |
| Moringa oil 1% | 4.34±0.05^a^ | 4.70±0.01^b^ | 5.07±0.01^b^ | 5.15±0.01^c^ | 5.40±0.01^a^ | 5.54±0.12^a^ | 5.43±0.02^a^ |
| Moringa oil 2% | 4.36±0.10^a^ | 4.70±0.04^b^ | 5.19±0.01^a^ | 5.24±0.02^b^ | 5.53±0.02^a^ | 5.74±0.12^a^ | 5.39±0.02^ab^ |
| Rosemary 1% | 4.54±0.21^a^ | 4.59±0.02^de^ | 4.75±0.02^f^ | 4.99±0.01^e^ | 5.29±0.03^a^ | 4.45±0.20^b^ | - |
| Rosemary 2% | 4.34±0.05^a^ | 4.71±0.02^b^ | 4.87±0.01^e^ | 5.12±0.02^cd^ | 5.44±0.01^a^ | 4.52±0.12^b^ | - |

The data were presented as mean ± SD (standard deviation). According to the Tukey test, means that do not share the letters for each variable in each column differ significantly at p≤ 0.05.
