# Supplementary material for: Sustainable chitosan and medicinal plant oils as natural edible coatings for postharvest quality preservation of guava fruits (Psidium guajava L.)
Source: PLoS One. 2026 Mar 18;21(3):e0342650. doi: 10.1371/journal.pone.0342650 (PMC12998884; doi:10.1371/journal.pone.0342650)
Supplement: S10 Table — (DOCX) [file pone.0342650.s010.docx]

**S10 Table**: Impact of chitosan and essential oils on non-reduced sugars (%) during cold storage conditions (at 8±1°C and 90±5% RH) of winter guava fruit ‘Etmany’ *cv*.

| treatment | Days after cold storage | | | | | | |
| --- | --- | --- | --- | --- | --- | --- | --- |
|  | 0 | 4 | 8 | 12 | 16 | 20 | 24 |
| control | 4.16±0.05^a^ | 4.73±0.01^a^ | 4.82±0.01^d^ | 5.27±0.01^a^ | 4.18±0.12^b^ | - | - |
| chitosan 1% | 4.21±0.15^a^ | 4.54±0.01^bc^ | 4.74±0.01^e^ | 5.03±0.02^c^ | 5.11±0.02^a^ | 4.25±0.12^b^ | - |
| chitosan 2% | 4.23±0.29^a^ | 4.50±0.02^cd^ | 4.82±0.01^d^ | 4.99±0.01^cd^ | 5.17±0.01^a^ | 5.40±0.02^a^ | 5.20±0.05^b^ |
| lemongrass oil 1% | 4.12±0.14^a^ | 4.45±0.01^de^ | 4.87±0.02^c^ | 4.96±0.03^d^ | 4.18±0.31^b^ | - | - |
| lemongrass oil 2% | 4.22±0.22^a^ | 4.49±0.01^cd^ | 4.98±0.01^b^ | 5.11±0.02^b^ | 4.18±0.31^b^ | - | - |
| Marjoram 1% | 4.36±0.25^a^ | 4.42±0.02^e^ | 4.50±0.02^g^ | 4.66±0.02^g^ | 4.26±0.17^b^ | - | - |
| Marjoram 2% | 4.05±0.13^a^ | 4.46±0.02^de^ | 4.66±0.01^f^ | 4.75±0.02^f^ | 4.25±0.31^b^ | - | - |
| Moringa oil 1% | 4.32±0.16^a^ | 4.57±0.01^b^ | 4.94±0.01^b^ | 5.02±0.01^c^ | 5.27±0.01^a^ | 5.41±0.12^a^ | 5.30±0.02^ab^ |
| Moringa oil 2% | 4.12±0.15^a^ | 4.57±0.04^b^ | 5.06±0.01^a^ | 5.11±0.02^b^ | 5.40±0.02^a^ | 5.61±0.12^a^ | 5.26±0.02^a^ |
| Rosemary 1% | 4.01±0.30^a^ | 4.46±0.02^de^ | 4.62±0.02^f^ | 4.86±0.01^e^ | 5.16±0.03^a^ | 4.32±0.20^b^ | - |
| Rosemary 2% | 4.39±0.09^a^ | 4.58±0.02^b^ | 4.74±0.01^e^ | 4.99±0.02^cd^ | 5.31±0.01^a^ | 4.39±0.12^b^ | - |

The data were presented as mean ± SD (standard deviation). According to the Tukey test, means that do not share the letters for each variable in each column differ significantly at p≤ 0.05.
