# Supplementary material for: Sustainable chitosan and medicinal plant oils as natural edible coatings for postharvest quality preservation of guava fruits (Psidium guajava L.)
Source: PLoS One. 2026 Mar 18;21(3):e0342650. doi: 10.1371/journal.pone.0342650 (PMC12998884; doi:10.1371/journal.pone.0342650)
Supplement: S11 Table — (DOCX) [file pone.0342650.s011.docx]

**S11 Table**: Impact of chitosan and essential oils on peroxidase (POD) enzyme activity (U/g) of fruits during cold storage conditions (at 8±1°C and 90±5% RH) of winter guava fruit ‘Etmany’ *cv*.

| treatment | Days after cold storage | | | | | |
| --- | --- | --- | --- | --- | --- | --- |
|  | 4 | 8 | 12 | 16 | 20 | 24 |
| control | 56.37±0.20^d^ | 42.34±0.17^f^ | 40.67±0.27^f^ | 36.14±0.15^f^ | - | - |
| chitosan 1% | 72.66±0.58^b^ | 73.67±0.32^c^ | 74.07±0.16^b^ | 74.33±0.30^b^ | 73.17±0.11^b^ | - |
| chitosan 2% | 73.67±0.53^b^ | 74.57±0.33^bc^ | 74.67±0.37^b^ | 75.67±0.55^ab^ | 74.37±0.68^a^ | 56.57±0.27c |
| lemongrass oil 1% | 73.57±0.36^b^ | 75.67±0.54^ab^ | 36.57±1.03^g^ | 16.67±0.38^i^ | - | - |
| lemongrass oil 2% | 73.27±0.31^b^ | 75.66±0.54^ab^ | 26.57±0.29^h^ | 16.65±0.46^h^ | - | - |
| Marjoram 1% | 65.67±0.42^c^ | 76.37±0.40^a^ | 36.55±0.28^g^ | 16.57±0.58^g^ | - | - |
| Marjoram 2% | 76.57±0.36^a^ | 65.87±0.43^e^ | 64.57±0.31^e^ | 46.57±0.46^e^ | - | - |
| Moringa oil 1% | 72.57±0.50^b^ | 73.67±0.33^c^ | 74.67±0.45^b^ | 76.47±0.30^a^ | 75.67±0.54^a^ | 65.33±0.59^b^ |
| Moringa oil 2% | 76.87±0.58^a^ | 75.67±0.54^ab^ | 76.27±0.34^a^ | 75.67±0.17^a^ | 74.57±0.45^ab^ | 70.07±0.61^a^ |
| Rosemary 1% | 72.67±0.50^b^ | 70.67±0.47^d^ | 71.57±0.30^c^ | 65.37±0.11^c^ | 54.66±0.43^c^ | - |
| Rosemary 2% | 72.67±0.38^b^ | 70.67±0.44^d^ | 69.87±0.44^d^ | 60.57±0.43^d^ | 50.27±0.69^d^ | - |

The data were presented as mean ± SD (standard deviation). According to the Tukey test, means that do not share the letters for each variable in each column differ significantly at p≤ 0.05.
