# Supplementary material for: Sustainable chitosan and medicinal plant oils as natural edible coatings for postharvest quality preservation of guava fruits (Psidium guajava L.)
Source: PLoS One. 2026 Mar 18;21(3):e0342650. doi: 10.1371/journal.pone.0342650 (PMC12998884; doi:10.1371/journal.pone.0342650)
Supplement: S12 Table — (DOCX) [file pone.0342650.s012.docx]

**S12 Table**: Impact of chitosan and essential oils on H_2_O_2_ content (µmol g^-1^ FW) of fruits during cold storage conditions (at 8±1°C and 90±5% RH) of winter guava fruit ‘Etmany’ *cv*.

| treatment | Days after cold storage | | | | | |
| --- | --- | --- | --- | --- | --- | --- |
|  | 4 | 8 | 12 | 16 | 20 | 24 |
| control | 30.65±0.34^a^ | 34.65±0.31^a^ | 40.65±0.12^a^ | 48.65±0.33^a^ | - | - |
| chitosan 1% | 27.65±0.18^b^ | 28.65±0.21^b^ | 26.65±0.32^e^ | 27.65±0.33^f^ | 28.65±0.33^c^ | - |
| chitosan 2% | 24.65±0.21^c^ | 25.65±0.32^de^ | 25.89±0.22^f^ | 26.65±0.21^g^ | 27.65±0.23^d^ | 28.85±0.21^a^ |
| lemongrass oil 1% | 24.03±0.07^cd^ | 26.65±0.18^c^ | 36.57±0.06^b^ | 42.32±0.12^b^ | - | - |
| lemongrass oil 2% | 23.65±0.27^de^ | 25.32±0.36^d^ | 30.32±0.09^d^ | 40.00±0.16^d^ | - | - |
| Marjoram 1% | 27.32±0.17^b^ | 28.65±0.35^b^ | 36.55±0.15^b^ | 42.32±0.17^b^ | - | - |
| Marjoram 2% | 24.65±0.35^c^ | 25.65±0.12^d^ | 34.35±0.12^c^ | 41.32±0.16^c^ | - | - |
| Moringa oil 1% | 23.05±0.27^e^ | 23.65±0.33^f^ | 24.65±0.33^h^ | 24.68±0.24^h^ | 24.89±0.17^e^ | 25.45±0.24^b^ |
| Moringa oil 2% | 22.65±0.24^f^ | 23.00±0.35^f^ | 24.02±0.02^h^ | 24.05±0.26^h^ | 24.25±0.07^e^ | 24.75±0.09^b^ |
| Rosemary 1% | 24.23±0.14^c^ | 24.32±0.31^e^ | 24.85±0.07^g^ | 30.75±0.13^e^ | 45.65±0.01^a^ | - |
| Rosemary 2% | 24.02±0.04^c-e^ | 23.32±0.19^f^ | 24.00±0.25^h^ | 30.57±0.07^e^ | 40.27±0.06^b^ | - |

The data were presented as mean ± SD (standard deviation). According to the Tukey test, means that do not share the letters for each variable in each column differ significantly at p≤ 0.05.
